# Six distinct NFκB signaling codons convey discrete information to distinguish stimuli and enable appropriate macrophage responses

**DOI:** 10.1016/j.immuni.2021.04.011

**Published:** 2021-05-11

**Authors:** Adewunmi Adelaja, Brooks Taylor, Katherine M. Sheu, Yi Liu, Stefanie Luecke, Alexander Hoffmann

**Affiliations:** 1 Institute for Quantitative and Computational Biosciences (QCBio), Molecular Biology Institute (MBI), and Department of Microbiology, Immunology, and Molecular Genetics (MIMG), University of California, Los Angeles (UCLA), 611 Charles E. Young Dr S, Los Angeles, CA 90093; 2 These authors contributed equally; 3 Lead contact

## Abstract

Macrophages initiate inflammatory responses via the transcription factor NFκB. The temporal pattern of NFκB activity determines which genes are expressed and thus, the type of response that ensues. Here, we examined how information about the stimulus is encoded in the dynamics of NFκB activity. We generated an mVenus-RelA reporter mouse line to enable high-throughput live-cell analysis of primary macrophages responding to host- and pathogen-derived stimuli. An information-theoretic workflow identified six dynamical features—termed signaling codons—that convey stimulus information to the nucleus. In particular, oscillatory trajectories were a hallmark of responses to cytokine but not pathogen-derived stimuli. Single-cell imaging and RNA sequencing of macrophages from a mouse model of Sjögren’s syndrome revealed inappropriate responses to stimuli, suggestive of confusion of two NFκB signaling codons. Thus, the dynamics of NFκB signaling classify immune threats through six signaling codons, and signal confusion based on defective codon deployment may underlie the etiology of some inflammatory diseases.

## In brief

Adelaja and Taylor et al. use a RelA-mVenus reporter mouse to examine, at single-cell level, the NFκB activation dynamics in primary macrophages responding to different stimuli. Their findings define six dynamical features—signaling codons—that classify immune threats and further suggest that signal “confusion” may contribute to autoimmune pathology.

## Graphical abstract

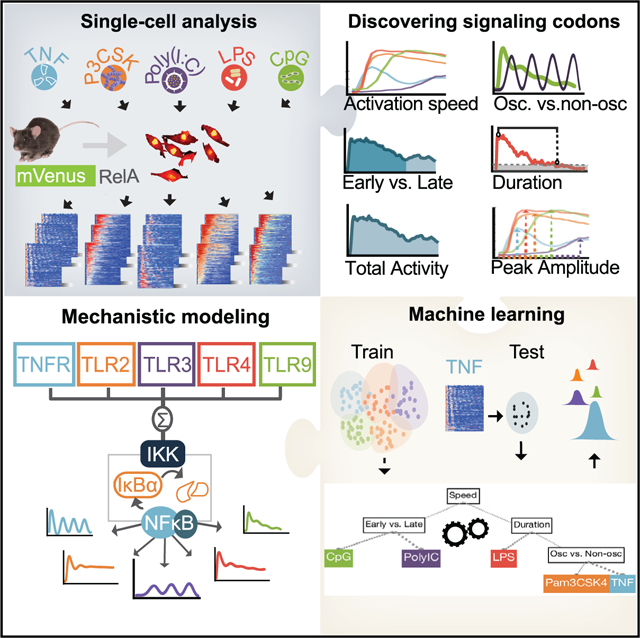

## INTRODUCTION

Autoimmune pathologies are characterized by the presence of auto-antibodies and immune attack of specific tissues, but the etiology is not uniform (Marshak-Rothstein, 2006). One cause may be found in errors in the negative selection of auto-reactive B cell or T cell clones in secondary lymphoid organs; another contributor may be inappropriate immune activation by immune sentinel cells (Marshak-Rothstein, 2006). Sjögren’s syndrome (SS) is a systemic autoimmune disorder that is characterized by progressive destruction of tissues exposed to the environment, such as eye, mouth and throat, and skin rashes (Marshak-Rothstein, 2006). Interestingly, genome-wide association studies do not point to salivary or lacrimal components (Burbelo et al., 2014), but rather to genes within the inflammatory pathways and immune cells (Taylor et al., 2017). Indeed, several genetic variants in regulators of the transcription factor NFκB are associated with SS patients (Lisi et al., 2012; Nordmark et al., 2013; Ou et al., 2008; Sisto et al., 2013), and a mouse strain containing similar variants recapitulates some of the SS pathognomonic characteristics (Peng et al., 2010). However, it remains unknown how these alleles affect NFκB dynamics.

Macrophages may function as immune sentinel cells that respond to pathogen invasion and tissue injury by initiating and coordinating both local and system-wide immunity (Wynn et al., 2013). These cells are ubiquitously distributed in tissues (Bauer et al., 2001) and can sensitively detect inflammatory cytokines and pathogen-associated molecular patterns (PAMPs), which indicate viral, bacterial, or fungal invasion (Medzhitov and Horng, 2009). Immune activation must be appropriate to each stimulus: the functional response to the cytokine TNF must be distinct from the response to a pathogen; further, the needs of a macrophage responding to bacterial or viral invasion are distinct.

The temporal coding hypothesis posits that information about the extracellular stimulus is represented in the time domain; i.e., the temporal pattern of a signaling activity (Behar and Hoffmann, 2010; Hoffmann and Baltimore, 2006; Purvis and Lahav, 2013). Biochemical studies in primary fibroblasts showed that the temporal pattern of NFκB RelA activity is stimulus specific at the cell population level (Covert et al., 2005; Werner et al., 2005), and that it controls the expression of immune response genes (Hoffmann et al., 2002; Tay et al., 2010). Although pioneering single-cell microscopy studies confirmed complex temporal patterns (Ashall et al., 2009; Nelson et al., 2004; Tay et al., 2010), they relied upon fluorescent-protein-NFκB RelA fusion proteins ectopically expressed in immortalized cell lines. Potential artifacts arising from ectopic expression of a reporter-effector protein have been reported (Barken et al., 2005; Cheong et al., 2011; Mothes et al., 2015), and prolonged cell culture adaptation of immortalized cell lines diminishes their responsiveness to immune threats (Cheng et al., 2015). These limitations have not allowed previous studies to explore the biological significance of temporal coding in primary immune cells and whether it is a useful concept for understanding immune pathology. Reasons for why no studies of single-cell NFκB trajectories in primary macrophages have been reported thus far include challenges associated with imaging proteins engineered to express fluorescent reporter constructs that are not overexpressed and reliable high-throughput image analysis of morphologically heterogeneous cells.

Here, we investigated the NFκB temporal code in single, primary macrophages using an mVenus-RelA mouse strain (Rela^v/v^) and a high-throughput imaging and analysis. An information-theoretic approach identified six dynamical features of the NFκB trajectories that convey information about the extracellular stimulus to the nucleus, which we term signaling codons. Teaching these to a machine demonstrated their sufficiency and requirement for ligand and dose identification. Indeed, examination of an SS mouse model revealed confusion of specific signaling codons and suggested that such confusion may contribute to the etiology of systemic autoimmune diseases. Finally, mathematical modeling allowed us to identify the molecular circuit design principles that enable encoding of these signaling codons and confirmed that “oscillations” are a hallmark of responses to the host cytokine TNF, in contrast to PAMPs transduced by the signaling adaptor MyD88.

## RESULTS

### Primary macrophages show immune threat ligand- and dose-specific NFκB dynamics

**Figure 1.**
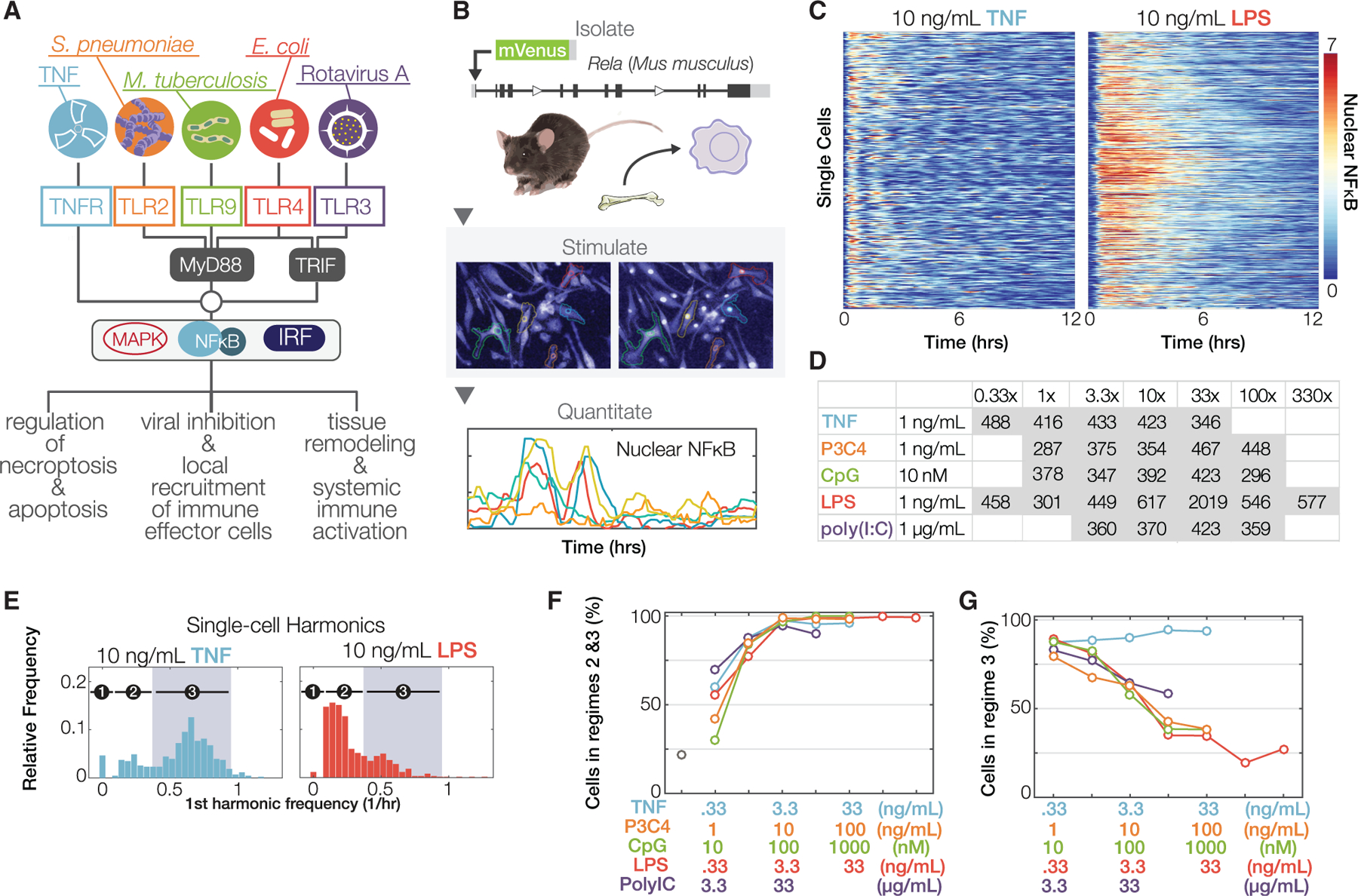
Complex NFκB dynamics induced by diverse immune threats (A) Schematic of the innate immune signaling network activating NFκB. Environmental information is transmitted via ligand-specific signaling pathways that converge on a few key transcription factors, including NFκB, but produce stimulus-specific physiological responses. (B) Workflow diagram: a reporter mouse line expressing mVenus-RelA (RelA^V/V^) was generated. Bone-marrow-derived macrophages (BMDMs) were differentiated, imaged, tracked, and quantified in multiple stimulus conditions. (C) Single-cell heatmaps of fluorescent nuclear NFκB levels over time, in BMDMs expressing endogenously tagged mVenus-RelA, in response to 10 ng/mL TNF or LPS. Each row is one cell’s NFκB trajectory. (D) Table indicating the number of single-cell NFκB trajectories quantified in each indicated experimental condition. This analysis involved 12,203 cell trajectories produced by quantifying more than 3 million cell images. More details in Table S2. All single-cell imaging data were confirmed, here and elsewhere, with at least two independent experiments per condition. (E) First-harmonic distributions for other stimuli. Shaded region corresponds to the period of 1–2.2 h that is characteristic of NFκB oscillations. (F) Fraction of cells in which a response is detected, by stimulus and dose. (G) Fraction of responder cells that show characteristic NFκB oscillations.

To extend pioneering research of NFκB dynamics in established cell lines (Table S1), we sought to study temporal patterns of nuclear NFκB in primary macrophages in response to prototypical immune threats (Figure 1A) at single-cell resolution. We generated the Rela^v/v^ mouse strain, which expresses a mVenus-RelA fusion protein (Figures S1A and S1B), similar to a previous GFP-RelA design (De Lorenzi et al., 2009) whose low fluorescence limited experimental studies (Sung et al., 2009). Macrophages, differentiated from primary bone-marrow cells derived from homozygous Rela^v/v^ mice, showed normal levels of nuclear NFκB binding activity (Figure S1C). Upon stimulation with a variety of different ligands and doses, and time-lapse imaging over 21 h (Figure 1B), the amount of nuclear NFκB fluorescence was quantitated in single cells using a fully automated image-processing pipeline that enabled tracking of live cells using minimal levels of a nuclear marker (Selimkhanov et al., 2014; Zambrano et al., 2016) and label-free identification and segmentation of cell cytoplasm. The live-cell imaging and image processing proved robustly reproducible in biological replicates (Figure S1D) and independent of image frame location (Figure S1E).

We noted differences in the NFκB dynamics induced by prototypical PAMP (LPS) and cytokine (TNF) stimuli, apparent at the single-cell level (Figure 1C). TNF induced oscillatory translocations between cytoplasm and nucleus that rapidly became desynchronized, matching biochemical data (Hoffmann et al., 2002). By contrast, LPS induced more than 4 h of sustained nuclear localization that also matched biochemical data from primary fibroblasts (Covert et al., 2005; Werner et al., 2005).

With an experimental workflow established, we recorded NFκB translocation dynamics in response to a large number of stimulation conditions, encompassing TNF and four different PAMPs, associated with diverse bacterial and viral pathogen classes (the TLR ligands CpG [TLR9], Pam3CSK4 [TLR1/2, referred to as P3C4], LPS [TLR4], and poly(I:C) [TLR3]) each tested at four to seven concentrations covering a 10^2^ to 10^3^-fold range. In each condition, 300–600 cells were examined with at least two preparations of BMDMs, thus constituting a total dataset of 12,203 single-cell trajectories captured with more than 3 million cell images and associated NFκB activity data-points (Figure 1D; Table S2).

Given the NFκB trajectory, each cell was classified based on its first harmonic frequency profile generated by Fourier analysis (Figure 1E) as either unresponsive (regime 1), responsive but non-oscillatory (regime 2), or oscillatory (regime 3) with a period of 1.1–2.2 h characteristic of NFκB oscillations (Hughey et al., 2015). Analysis of the data indicated that the lowest stimulus concentration activated about half the cells but that a log_10_ increase activated almost all (Figure 1F). Plotting the percentage of cells classified as oscillators in responders, we found that the host factor TNF elicited oscillatory dynamics regardless of dose (Figure 1G). While the number of peaks increased with increasing doses of TNF, the period remained constant (Figure S1F). In contrast, PAMPs produced largely non-oscillatory responses at high ligand concentrations (Figure 1G). Unlike experimental systems with ectopically expressed RelA, which produced a first peak of NFκB activity that is much higher than later peaks (Ashall et al., 2009; Hughey et al., 2015; Tay et al., 2010), the response of primary macrophages to TNF showed a constant, gradual fall-off in amplitude (Figure S1G). Representative trajectories from various stimulus conditions indicate that NFκB dynamics are ligand and dose specific (Figure S1H).

### Informative dynamical features are identifiable

**Figure 2.**
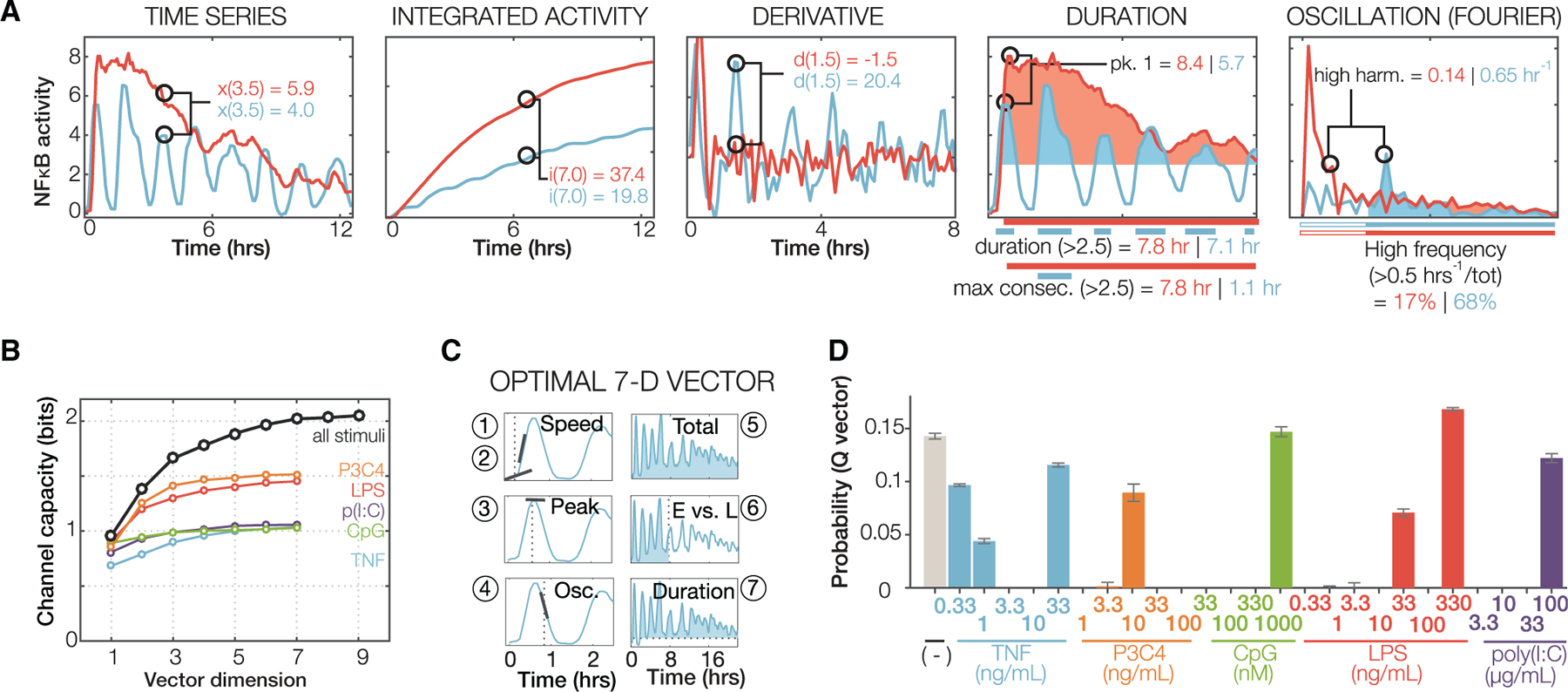
Informative features within complex NFκB dynamics (A) Examples of metrics to be employed in an information theoretic analysis. Two single-cell NFκB responses (to LPS in red, and to TNF in blue) are shown. All NFκB trajectories were characterized using 918 metrics (Table S3). (B) Channel capacity as a function of the number of most informative metrics (Table S4), either using the entire dataset of all ligand types and doses (black line) or using the dose response data for each indicated ligand. Channel capacity is a correlation score based in information theory; it indicates the degree to which a metric of NFκB dynamics or a combination of such metrics are correlated with the stimulus condition, defined by ligand identity and dose. (C) Dynamical features that are informative about ligand and dose, as revealed by the seven metrics selected by the information theoretic analysis. E: early activity; L: late activity. (D) Average probability distribution from the channel capacity calculations using all optimal vectors. Probabilities sum to 1 and indicate the input distribution that leads to a computationally maximized mutual information.

Oscillations are just one dynamical feature by which complex time course trajectories can be characterized. We developed a method for identifying dynamical features that are associated with stimulus- and dose-specific NFκB trajectories. We constructed a multivariate information-theoretic algorithm, based on an estimate of channel capacity (Cheong et al., 2011; Selimkhanov et al., 2014). In conjunction with the primary timeseries data, we considered 918 derived metrics (Table S3) such as integrals, derivatives, peak activities, durations, or frequencies (Figure 2A). Our algorithm searched this library for combinations of metrics that maximized channel capacity (Figure S2A), iteratively expanding the number of metrics within each combination from two up to ten.

First, we considered the available dose response dataset for each ligand separately. Combinations of five metrics were sufficient to capture the mutual information of dose responses, with TNF, CpG, and poly(I:C) achieving about 1 bit and LPS and Pam3CSK4 about 1.5 bits (Figure 2B), in agreement with previous reports for TNF and LPS (Cheong et al., 2011; Selimkhanov et al., 2014). When considering all ligands tested (26 dose-ligand conditions), the calculated channel capacity was markedly higher (>2 bits) and required a seven-dimensional vector to yield ≥95% of the maximum measured information content (Table S4).

Of these most informative metrics identified across the full dataset (Figure 2C), two defined the activation speed (1), one defined the peak amplitude (2), another defined the post-induction repression, a distinguishing feature of oscillatory versus non-oscillatory trajectories (3), one defined the accumulated activity (integral) at a late time (4), one was a measure of the degree to which NFκB activity is “front loaded” (5), and one defined the total duration of NFκB activity above a low threshold (6). Thus, the information-theoretic analysis identifies six NFκB dynamical features that are informative about the stimulus ligand and dose. Plotting three features allowed for only incomplete separation of ligands (Figure S2B).

Further analysis of the channel capacity calculations indicated that the highest dose generally provided the most ligand-specific information (Figure 2D). Indeed, when we restricted the calculation to only the highest dose of each of our five ligands, we still obtained a channel capacity of 1.86 bits. Unlike the dose response profiles of pharmacological agents that tend to show cross-reactivity at high doses, ligand-specific signaling dynamics occur at highest doses, indicating that there are true differences in the signal processing characteristics of receptor-associated signaling pathways.

### Machine learning of NFκB codons distinguishes stimuli

**Figure 3.**
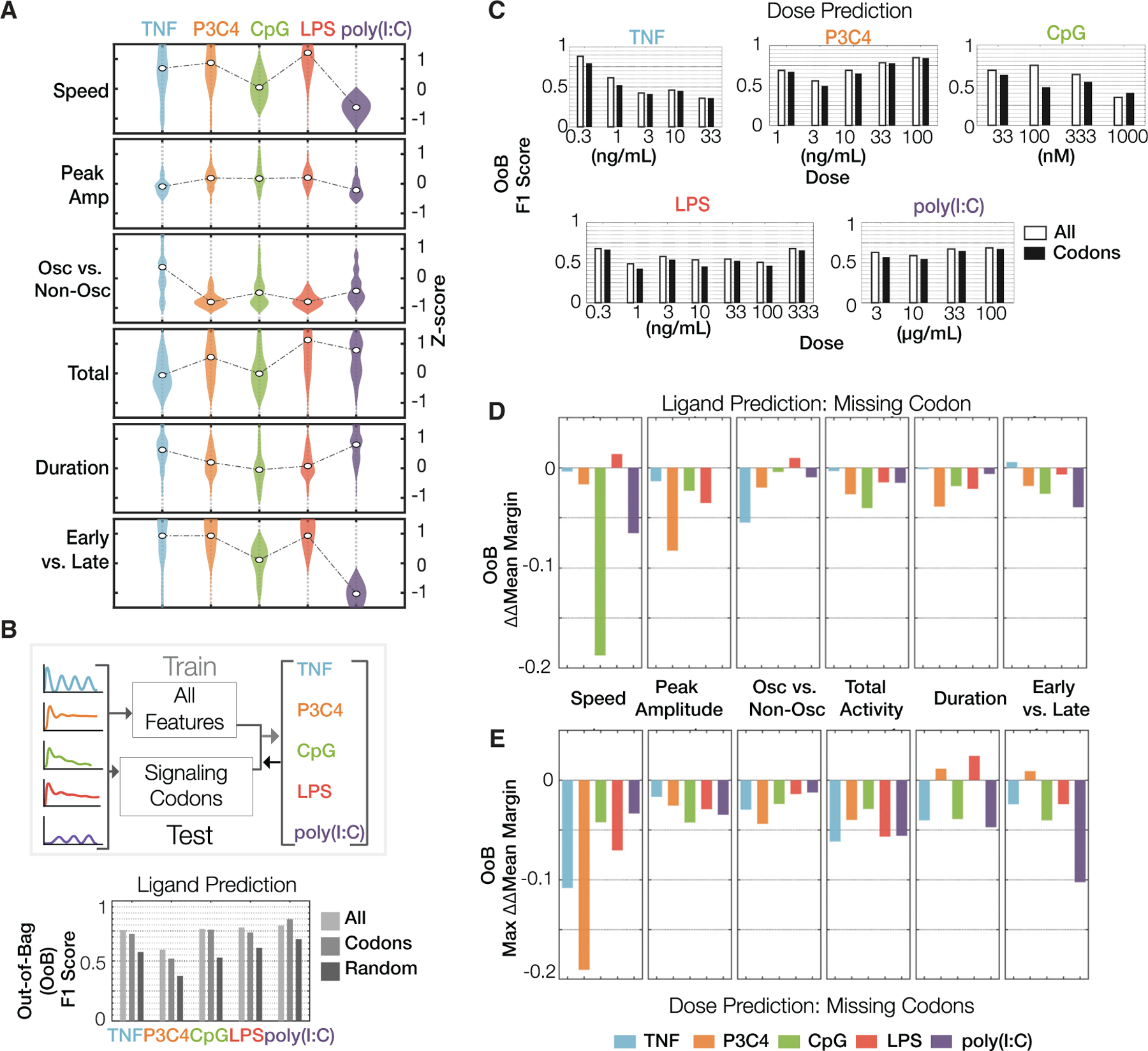
Six NFκB signaling codons are sufficient to classify immune threats (A) Violin plots of dynamical features that optimally encode stimulus-specific NFκB dynamics: activation speed, peak amplitude, oscillatory dynamics, total activity, duration, and ratio of early to late activity. These are termed “signaling codons,” and they are deployed in a stimulus-specific manner, as shown. (B) Top: schematic of supervised machine learning approach to predict ligand identity using NFκB dynamics. Bottom: F1 scores (harmonic mean of precision and recall) of ligand predictions using either all features or signaling codons alone or random. Models are evaluated on out-of-bag observations. (C) F1 score of dose predictions for each indicated ligand using either all features or only six signaling codons. (D) The effect of each signaling codon on the certainty of ligand prediction: the loss in classification confidence when the indicated signaling codon is missing from the set of six (versus all features). Mean classification margin: probability of the correct class minus the highest probability of the incorrect classes; ΔMean Margin: difference in mean classification margin of codon classifier versus all predictors classifier; ΔΔMean Margin: difference in ΔMean Margins when using a classifier with all six signaling codons and with classifiers lacking the indicated signaling codon. (E) The effect of each signaling codon on the certainty of dose prediction for each ligand: the loss in classification confidence when the indicated signaling codon is missing from the set of six (versus all features).

The six NFκB dynamical features, identified as conveying information about the extracellular stimulus to the nucleus, represent potential codewords of the temporal NFκB signaling code and are referred to as NFκB signaling codons. Visualizing signaling codon deployment for the five ligands at high doses (Figure 3A; Table S5), the speed of activation is generally high for Pam3CSK4 and LPS-triggered signaling, but low for CpG and poly(I:C) and inter-mediate for TNF; peak amplitude is high for Pam3CSK4, CpG, and LPS and lower for TNF and poly(I:C); the oscillatory content is highest for TNF compared to any of the PAMPs; the amount of total activity is highest for LPS followed by poly(I:C) and Pam3CSK4, but lower for TNF and CpG; the total duration, in contrast, is high for TNF and poly(I:C) and relatively low for Pam3CSK4, CpG, and LPS; and the fraction of the activity that is early is much higher for TNF, Pam3CSK4, and LPS than poly(I:C), with CpG being inter-mediate. Similarly, we find that different doses of the same ligand may deploy the signaling codons differentially (Figure 3A). For example, while the peak activity is generally positively correlated with dose (Lee et al., 2014a), the duration of activity increases with increasing doses of TNF or LPS but decreases with increasing doses of CpG.

To determine whether the six NFκB signaling codons suffice to distinguish these ligands, we used supervised machine learning and trained an ensemble-of-decision-trees model either with all 918 metrics or the set of 6 signaling codons (Figure S3B). We chose this classification algorithm because of its performance and interpretability (Alpaydin, 2014; Caruana and Niculescu-Mizil, 2006). Assessing prediction performance, we found that F1 scores (harmonic mean of precision and sensitivity, a measure of specificity and sensitivity of the predictions) were remarkably similar for predictions generated using all metrics or just signaling codons, while the average of randomly sampled features fared substantially worse even when optimally trained (Figure 3B; Table S6A). Other performance measures confirmed this conclusion (Figure S3C), indicating that six signaling codons suffice to distinguish NFκB ligands. Using the same approach, we examined whether signaling codons suffice to distinguish the doses of each ligand (Figure 3C; Table S6B). The differences in F1 scores of dose predictions generated by classifiers trained using all features versus six signaling codons were minimal.

We quantified the certainty or level of confidence of stimulus classification (classification margin; the probability assigned to the correct class minus the highest probability assigned to any of the incorrect classes) using all features, signaling codons, and subsets of signaling codons (Figure 3D). To examine the necessity of each signaling codon, we computed ΔΔMean Margin, which is the difference between the quantities obtained by (1) normalizing the mean classification margin obtained from six signaling codons by subtracting the mean classification margin obtained from all features to compute ΔMean Margin, and (2) normalizing the mean classification margins from all combinations of five signaling codons (all subsets where only one codon is missing). We used ΔΔMean Margin to interrogate the necessity of each signaling codon by computing the normalized difference in mean classification margin in the presence (set of six codons) and absence of each codon (all sets of five codons). This analysis revealed the stimulus-specific dependence of the classification certainty on each signaling codon: speed is important in classifying CpG and poly(I:C), peak amplitude is important for classifying Pam3CSK4, and oscillatory dynamics are important for classifying TNF (Figure 3D).

To examine the necessity of each signaling codon in distinguishing doses, we quantified the ΔΔMean Margin across all doses for each ligand (Figures 3E, S3E, and S3F). The maximum ΔΔMean Margin across all doses of each ligand revealed that speed is important to distinguish doses of TNF, Pam3CSK4, and LPS, and “early versus late” activity is important to distinguish doses of poly(I:C). Furthermore, this analysis suggests that the importance of a signaling codon for classifying a ligand may differ from its importance in distinguishing the doses of that ligand (Figures 3D and 3E). Using binary classification of stimulated condition versus vehicle control indicated that ligand identification increases with the dose of the stimulus (Figure S3G), confirming the results of the information theoretic analysis (Figure 2D).

### Increased signaling codon confusion in an autoimmune disease model

**Figure 4.**
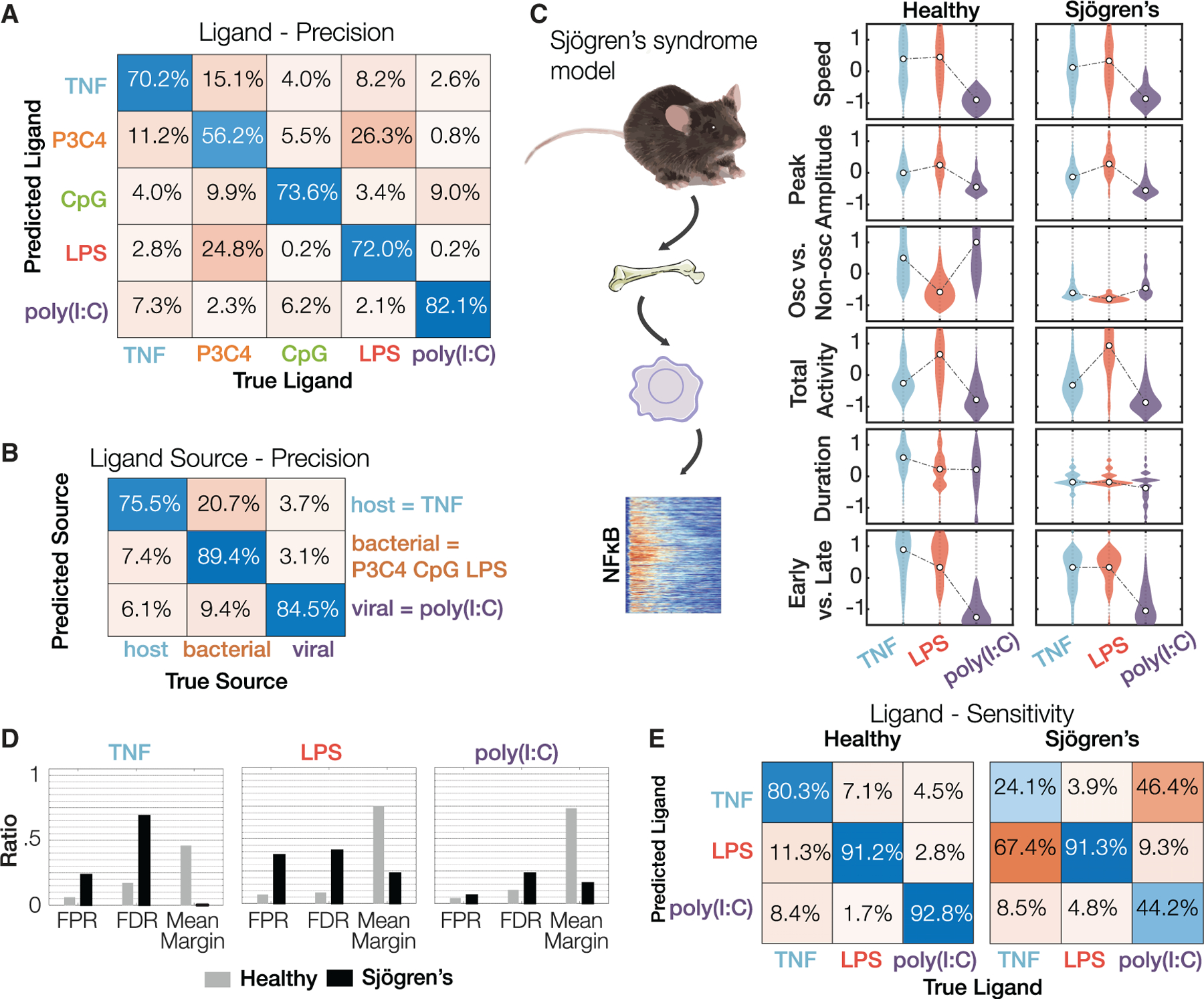
A Sjögren’s syndrome mouse model shows more confusion in classifying immune cytokine TNF and immune threat LPS based on NFκB dynamics (A) Confusion matrices showing classification precision of ligand identity information. The machine learning model correctly identifies the ligand identity given an NFκB trajectory a majority of the time with the primary confusion being between bacterial ligands Pam3CSK4 and LPS most apparent. Evaluated by 5-fold cross-validation. (B) Confusion matrices showing classification precision of ligand source information. Bacterial ligands are generally correctly identified as such. Evaluated by 5-fold cross-validation. (C) Testing ligand confusion in macrophages isolated from a Sjögren’s disease model mouse (Peng et al., 2010). Violin plots depicting the signaling codons deployed by macrophages, derived from healthy or Sjögren’s mice, stimulated with TNF, LPS, or poly(I:C). (D) Classification of ligand identity in healthy and Sjögren mouse model macrophages by a machine learning classifier trained on healthy macrophage data: false positive rate (FPR), false discovery rate (FDR), and mean margin. Evaluated by 5-fold cross-validation and an independent test set (Figure S4). (E) Confusion matrices for sensitivity/recall for the healthy and Sjögren’s macrophage data. Evaluated by 5-fold cross-validation and an independent test set (Figure S4).

The availability of a validated machine learning classifier allowed us to quantify not only how precise stimulus identification is, but which other stimuli a given stimulus may be confused with. We characterized the points of confusion by quantifying classification accuracy (precision) in the matrix of five ligands, choosing their highest doses as they are most distinguishable (Figures 4A, S4A, and S4B). Correct classification of ligand identities occurred in the majority, but misclassifications (off-diagonal values) were not uniformly distributed. For example, while confusion of viral PAMP poly(I:C) and bacterial PAMP LPS was rare, it was more common between the bacterial PAMPs, LPS, and Pam3CSK4. Indeed, when we grouped ligands into their source classes such as host (cytokine), bacteria, or virus, we found that bacteria-derived ligands are reliably distinguished and show little confusion with either virus- or host-derived ligands (Figures 4B and S4D). To assess the dependence of classification performance on the number of trajectories, we sub-sampled the number of trajectories and evaluated the precision and sensitivity of classification (Figures S3H–S3L). This analysis revealed that performance reached saturation with just 50% of the data used in the original model training. Further, we compared the classification performance of signaling codons to time series data, and time series data transformed by an autoencoder: we found that signaling codons performed as well as time series data (Figure S3K) and fared substantially better than autoencoder-transformed time series data (Figure S3L).

We assessed whether a mouse model of SS (Peng et al., 2010), which mimics genetic variants of the regulatory region of the NFκB regulator IκBα found in human patients (Lisi et al., 2012; Nordmark et al., 2013; Ou et al., 2008; Sisto et al., 2013), may be associated with signaling codon confusion, such that cells exposed to one stimulus might in fact miscommunicate the presence of a different stimulus to nuclear target genes. We bred our mVenus-RelA reporter into this mouse model and then derived bone-marrow-derived macrophages for stimulation with the cytokine TNF, the bacterial PAMP LPS, and the viral PAMP poly(I:C) (Figure 4C). Unlike macrophages from healthy mice, these SS macrophages showed non-oscillatory NFκB trajectories in response to all stimuli (Figure S4E). Visualizing the distributions of the six signaling codons revealed that the stimulus-specific deployment of particular NFκB signaling codons was impaired in macrophages from the Sjögren’s mouse model (Figure 4C). The stimulus specificity of the “oscillatory” codon was markedly diminished in SS macrophages, and the stimulus specificity of the “duration” and the “early versus late” codon was also affected.

Then, we examined the accuracy of stimulus classification using the ensemble-of-decision-trees algorithm (Table S6C). The mean margin scores of ligand classification were greatly diminished in SS macrophages, concomitant with an elevation in the false positive and false discovery rates for TNF and LPS (Figure 4D). Furthermore, the sensitivity of TNF and poly(I:C) classification in SS macrophages was greatly diminished (24.1%/44.2%, respectively, versus 80.3%/92.8% in healthy controls, Figure 4E), as there is increased confusion between poly(I:C) versus LPS, and TNF versus LPS. Similarly, the precision of TNF and LPS classification was greatly diminished (31.1%/58.9%, respectively, versus 83%/91.5% in healthy controls, Figure S4F). These analyses indicate that SS macrophages have diminished ability to generate stimulus-specific NFκB signaling dynamics and suggest that signaling codon confusion and mistranslation may play a role in the etiology of sporadic inflammatory diseases.

### NFκB signaling codon confusion diminishes the stimulus specificity of gene expression

**Figure 5.**
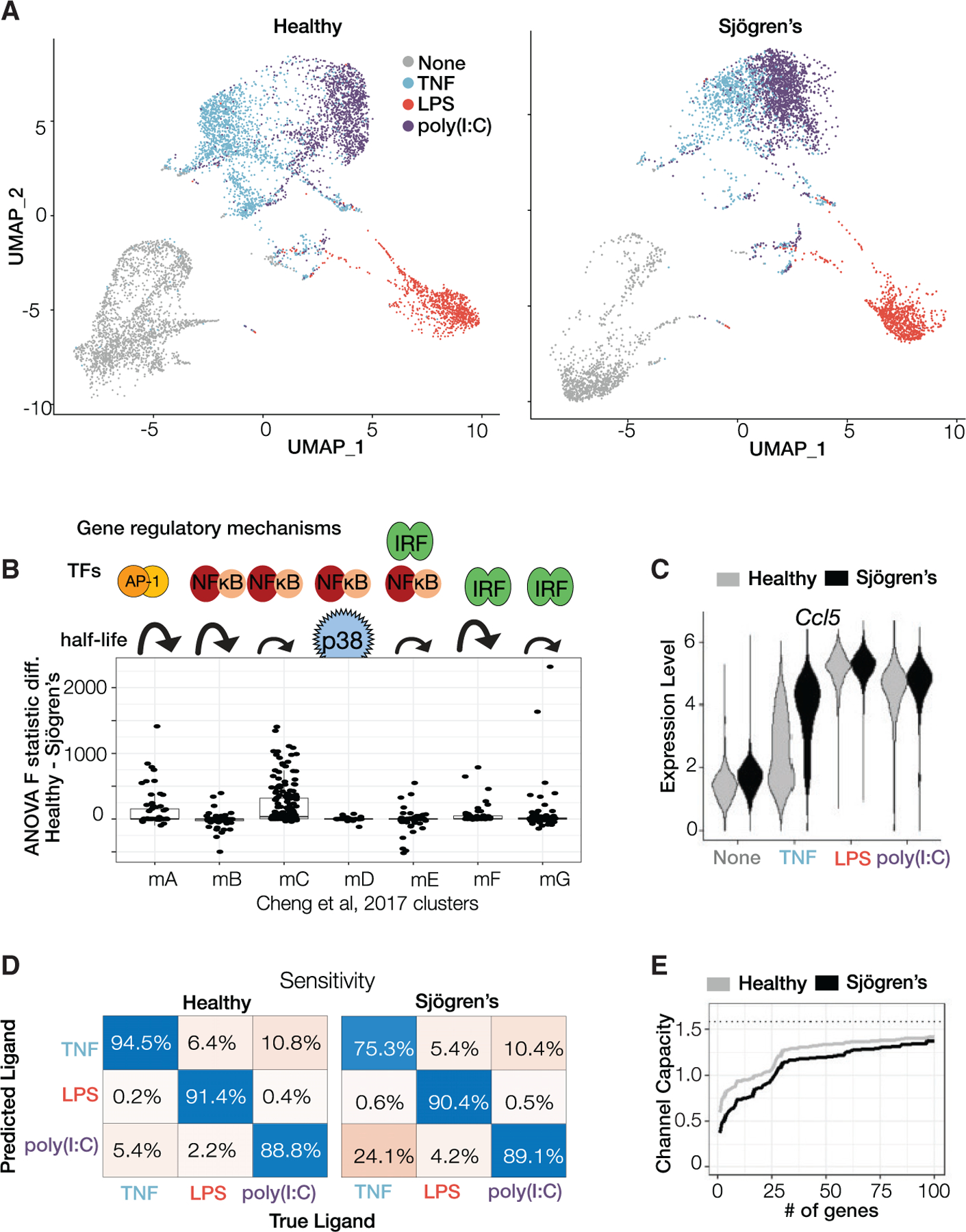
Stimulus specificity of gene expression responses is diminished in macrophages from a Sjögren’s mouse model (A) Single-cell RNA sequencing data of healthy and SS BMDMs collected after 8 h of stimulation with indicated ligands is visualized using the UMAP dimensionality reduction technique. (B) Genes plotted by loss of stimulus specificity (difference of ANOVA F statistic between healthy and SS) in expression, grouped by the indicated gene regulatory clusters identified in Cheng et al., 2017. Positive difference represents greater stimulus specificity in healthy than in SS. (C) Violin plots depicting the expression of *Ccl5* in individual cells stimulated in indicated conditions. (D) Confusion matrices from a random forest classifier comparing the distinguishability (sensitivity/recall, a measure of accuracy) of each ligand between healthy and SS. The classifier was trained on top 100 genes and was evaluated using a 30% holdout set. (E) Comparison of channel capacity (the maximum amount of information about ligand identities that can be abstracted from expression of genes; Mackay, 2003) as a function of the number of genes between Healthy and SS cells. Genes were added by forward selection based on ANOVA F statistic difference ranking. Dotted line represents theoretical maximum for three stimulus conditions.

We wondered whether the diminished specificity of NFκB activation dynamics affected the stimulus specificity of downstream gene expression. To this end, we stimulated macrophages as before but subjected them to single-cell RNA sequencing (scRNA-seq, using the 10X genomics platform) after 8 h, reasoning that gene expression follows transcription factor activation. After normalizing counts to library size and log transforming, we performed principal-component analysis (PCA) on *Z*-scored data and displayed the data on two-dimensional UMAP plots using the top 20 principal components (Figure 5A). In healthy macrophages, expression clusters were readily distinguishable between unstimulated cells and cells stimulated with TNF, LPS, or poly(I:C). However, in SS cells, the distinction between TNF and poly(I:C) was slightly diminished.

We asked which genes may be affected in their expression specificity. We performed ANOVA (Figure S5A) and calculated channel capacity (Figure S5B) for each individual gene using the three stimulus conditions to determine genes that lose specificity in SS macrophages versus control. These two calculations provided a reasonably concordant ranking of genes contributing to differences in expression specificity in healthy versus SS macrophages (Figure S5C). Leveraging the pathway-target gene mapping of Cheng et al., 2017, we found that NFκB target genes with long mRNA half-lives were especially strongly affected in their stimulus specificity (Figure 5B). One example gene ranked highly in loss of stimulus specificity is *Ccl5*, which shows a high degree of heterogeneity in expression in response to TNF, being expressed highly in a minority of cells in healthy macrophages, but in the vast majority of SS macrophages (Figure 5C). Thus, *Ccl5* expression in SS macrophages is less distinguishable in whether it is induced by TNF or poly(I:C).

To characterize the overall stimulus-response specificity of macrophages, we used the top 100 differentially expressed genes ranked by difference in ANOVA F statistic (Figure S5A) to train a random forest classifier with 10-fold cross-validation using 70% of single cells from the healthy macrophage population. Testing the trained model on the remaining held-out data demonstrated that healthy macrophages distinguish between the three stimuli with >90% sensitivity, but testing the model on SS macrophages revealed that >20% of SS macrophages produced indistinguishable gene expression programs in response to TNF and poly(I:C) (Figure 5D). Concordant with the machine learning results, top differentially expressed genes in healthy cells showed greater confusion between TNF and poly(I:C) (Figure S5D), with the classification false positive rate for TNF being about five times higher in SS cells than healthy cells (Figure S5E). In contrast, LPS-induced gene expression remained distinguishable, presumably because the LPS-specific MAPKp38 pathway provides for several LPS-specific induced genes regardless of altered NFκB dynamics (Figure 5B). The reduction in channel capacity was robust to subsampling the number of cells for which we had data (Figure S5F). Interestingly, the confusion was driven by the loss of stimulus-specific information associated with dozens of genes, as SS macrophages performed almost as well as healthy controls when considering just 100 genes (Figure 5E). Examining the genes that are more specific in healthy than in SS macrophages revealed gene ontology terms such as innate immune response (Figure S5G) and IRF binding motifs (Figure S5H) that are enriched in their regulatory regions. This suggests that the confusion of NFκB signaling oscillatory and duration codons diminishes the stimulus specificity of interferon stimulatory genes (ISGs) via the inappropriate expression of type I interferon. Type I interferon has long been associated with Sjögren’s pathologies (Marketos et al., 2019; Muskardin and Niewold, 2018).

### Molecular circuits that produce signaling codons

**Figure 6.**
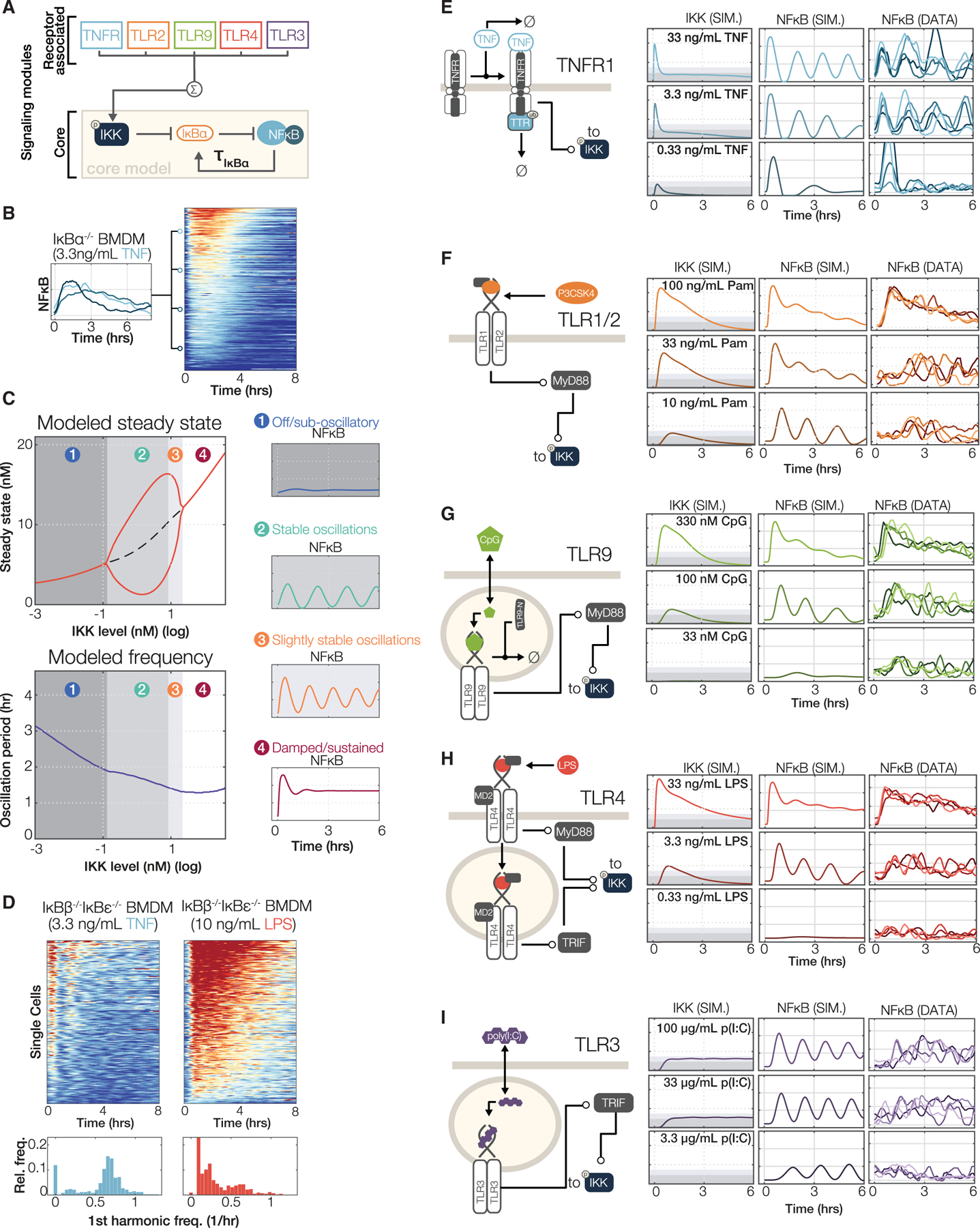
Kinetic models of receptor-associated signaling modules share circuit design principles that generate NFκB signaling codons in a stimulus-specific manner (A) A simple schematic suggesting that NFκB control is mediated by two regulatory networks: the core IκBα-NFκB signaling module is downstream of receptor-associated signaling modules. Receptor-associated signaling modules determine IKK activity over time. Within the core module, IKK activity destabilizes IκBα, freeing NFκB to translocate to the nucleus, where it induces expression of IκBα. (B) The IκBα-feedback is required for generating the oscillatory component of NFκB dynamics characteristic of the response to TNF. Single-cell trajectories and heatmaps of NFκB responses to 3.3 ng/mL TNF in BMDMs derived from RelA^V/V^, IκBα-deficient mouse. (C) A mathematical model predicts bifurcating behavior in NFκB dynamics based on the level of IKK activation. Left: model steady-state values and primary oscillation frequency are shown as a function of sustained IKK level (Hopf bifurcation analysis). Right: single simulated trajectories of IKK and NFκB activation, at each of four regimes identified in the steady-state diagram. (D) The IκBα feedback loop is sufficient to sustain the non-oscillatory characteristic of the NFκB response to LPS. Single-cell heatmaps of NFκB responses to 3.3 ng/mL TNF and 10 ng/mL LPS in BMDMs derived from a RelA^V/V^IκBβ^−/−^IκBε^−/−^ mouse. Below each heatmap, a histogram indicates each cell’s first harmonic showing relative proportions of oscillatory cells (n > 400 individual cells for each experiment, representative of two independent replicates). (E–I) Simplified schematics showing salient features of TNF, TLR1/2, TLR9, TLR4, and TLR3 signaling pathways, and the simulated IKK and NFκB activity (left/middle) and four measured median cell NFκB trajectories (right) at each of three log-spaced (TNF and TLR4) or four half-log-spaced (TLR9, TLR1/2, and TLR3) doses of each receptor’s cognate ligand. The complete reaction sets of the model are described in STAR Methods and Table S7.

Having identified essential dynamical features of NFκB activity for encoding ligand identity and dose, we sought to understand the molecular mechanisms that provide for the diversity of stimulus-specific dynamics. The known topology of the NFκB network is that signals emanating from receptor-associated signaling modules converge to activate canonical IKK, which functions as the input to the IκB-NFκB signaling module whose most prominent regulator is IκBα (Figures 6A and S6A). A prominent signaling codon that distinguishes the cytokine TNF from PAMPs is the oscillatory content. Using macrophages from an IκBα-deficient mouse (interbred with the mVenus-RelA reporter, see STAR Methods), we found at the single-cell level that oscillatory dynamics are dependent on IκBα negative feedback (Figure 6B), in agreement with prior population level experiments (Fagerlund et al., 2015; Hoffmann et al., 2002).

Then, we asked whether the IκBα feedback loop may also mediate non-oscillatory responses characteristic of PAMPs or whether other IκB isoforms may be required. After adapting the mathematical model of the negative-feedback containing IKK-IκBα-NFκB signaling module to the primary macrophage (see STAR Methods), we examined its dynamical properties using Hopf-bifurcation analysis, specifically the propensity for oscillatory responses as a function of the magnitude of IKK activity (Figure 6C). The first bifurcation point defines the threshold between (1) “off” (indistinguishable from baseline activity) and (2) an oscillatory steady-state. As IKK activity increases, oscillation troughs rise in amplitude (3) though the period changes little. The second bifurcation point occurs as the system shifts to highly damped oscillations (4). Our analysis thus led to the prediction that non-oscillatory NFκB responses of LPS are not mediated by other IκB isoforms (IκBβ and IκBε), as previously hypothesized (Kearns et al., 2006; Thompson et al., 1995), but that the NFκB-IκBα feedback circuit alone could sustain such non-oscillatory behavior. To test this hypothesis, we bred our RelA-mVenus reporter into *IkBb*^−*/*−^*IkBe*^−*/*−^ mice and measured single-cell responses to TNF and LPS (Figure 6D). In this genotype, TNF induced an even higher fraction of oscillatory cells (95% versus 75% in wild-type, Figure 1G), while LPS responses were, as before, largely non-oscillatory. We conclude that both oscillatory and non-oscillatory NFκB dynamics may be generated by the IκBα-NFκB signaling module; the deployment of the oscillatory signaling codon is determined merely by controlling the amount of IKK activity over time.

To build a full, multi-stimulus model capable of generating proper IKK activity time courses in response to any of the ligands and doses used in this study, we carefully examined the regulatory mechanisms associated with each ligand receptor (Figure S6A) and drafted ordinary differential equations to describe them. Parameter values were based on prior literature (Table S7) and adjusted to produce model simulations of NFκB that qualitatively matched trajectories of median-responding cells in each tested condition (Figures 6E–6I). For TNF and LPS, available literature datasets on receptor and IKK dynamics were fit (Figures S6B and S6C). Within the core IKK-IκB-NFκB module, multi-parameter sampling confirmed that the oscillatory-non-oscillatory distinction based on the magnitude of IKK activity was a robust feature (Figure S6D). This conclusion was further supported by the observation that when macrophages are costimulated with oscillation-producing TNF and the non-oscillatory dynamics producing CpG, the resulting NFκB trajectories are non-oscillatory (Figure S7A). Quantitative analysis of these data revealed that the distributions of the “oscillatory,” “duration,” “speed,” and “early versus late” codons are nearly indistinguishable in response to CpG + TNF and CpG alone, though they are distinguishable in response to TNF alone (Figure S7B). In addition, model-simulated IKK trajectories (Figures 6E–6I) were tested at key time points using immunoblotting of the active, phosphorylated IKK species (Figures S7C–S7G) and revealed a general concordance in this semiquantitative comparison. While this increases our confidence in the insights derived from the model, we cannot rule out alternative models or mechanisms.

Signaling within each signaling module is governed largely by the kinetic properties of a few constituents such as ligand half-life, receptor downregulation and replenishment, and the dose response properties of the receptor-associated signaling adaptor. For example, in the case of TNF, rapid receptor downregulation and short ligand half-life (Cheong et al., 2006; Werner et al., 2008) diminish IKK activity into a regime that allows for deployment of the “oscillatory” codon and the dose-dependent deployment of the “duration” codon, respectively (Figure 6E). For Pam3CSK4 and CpG (Figures 6F and 6G), the signaling characteristics of cooperative adaptor interactions lead to digital dose response behavior (Cheng et al., 2015) and low values for the “oscillatory” (due to high IKK activity) and “duration” codons at high doses. In the case of LPS-TLR4 (Figure 6H), the combination of ultrasensitive and linear dose response behavior of MyD88 and TRIF adaptors (Cheng et al., 2015; Kellogg et al., 2015), aided by CD14-mediated TLR4 internalization (Zanoni et al., 2011), provide for dose-dependent deployment of the “oscillatory” and “total activity” signaling codons. In contrast, endosomal availability of TLR3 and poly(I:C) (O’Mahony et al., 2008) limit the “response speed” codon but allow for long duration (Figure 6I). Overall, the comparison of five signaling modules revealed shared molecular circuit design principles whose pathway-specific parameter values yield diverse ligand- and dose-specific deployment of NFκB signaling codons.

### Oscillatory NFκB dynamics are a hallmark of paracrine TNF signaling

Overall, model simulations qualitatively matched measured trajectories at the respective doses. However, we identified a notable discrepancy in the responses of the MyD88-dependent pathway downstream of TLR9 at low doses (33 nM CpG, Figure 6G). Simulations in this condition did not show substantial NFκB activation, but the measured trajectories showed oscillatory responses.

**Figure 7.**
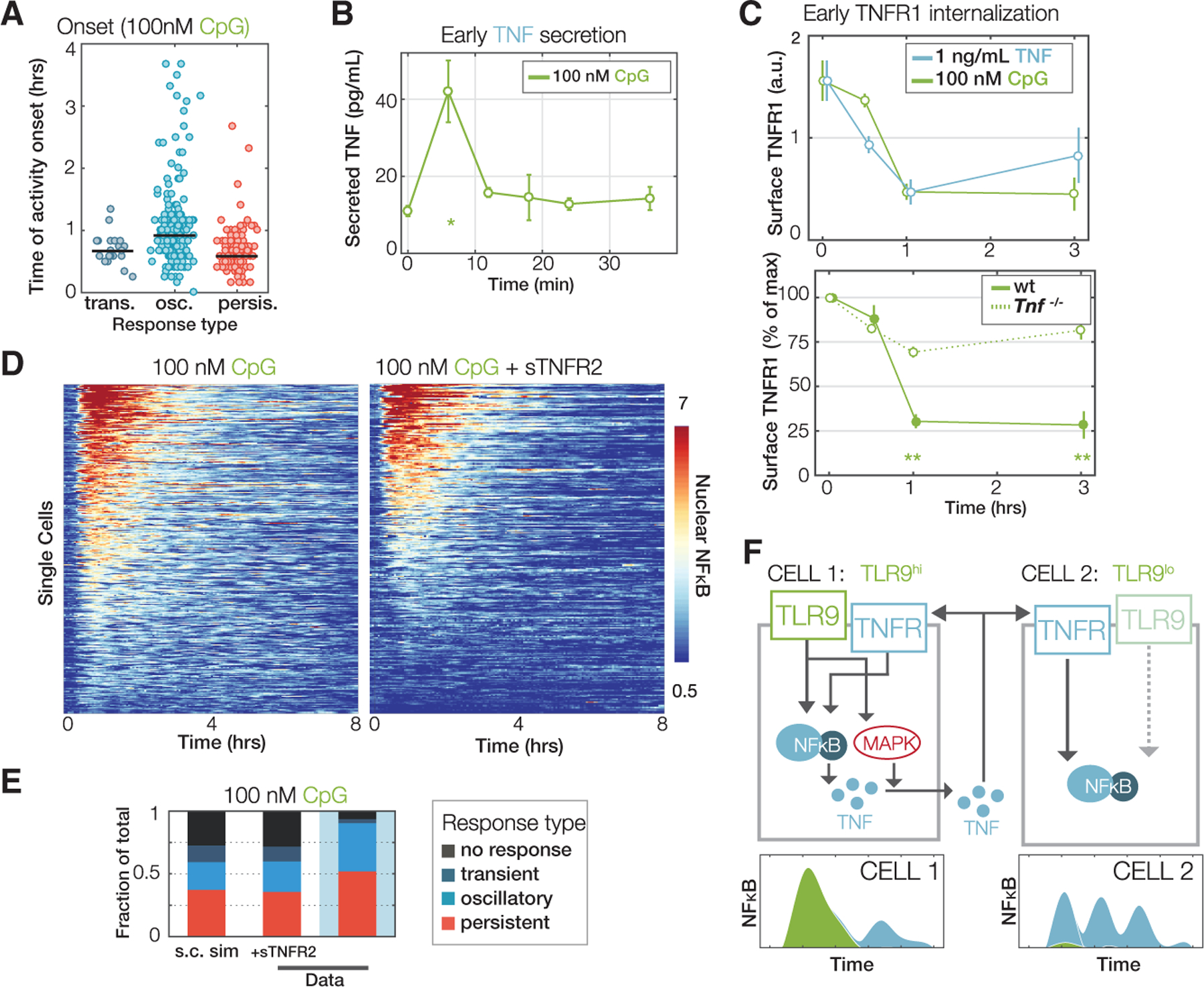
Oscillatory NFκB in response to PAMPs is a hallmark of feedforward TNF (A) Activity onset times in single-cell NFκB responses to 100 nM CpG, grouped by dynamic subtypes of the response (persistent, oscillatory, or transient). (B) Early-phase TNF secretion dynamics from macrophages stimulated with 100 nM CpG, as measured by ELISA. (C) Top: median surface TNFR1 expression over time in BMDMs exposed to 1 ng/mL TNF or 100 nM CpG, monitored by flow cytometry. Bottom: median surface TNFR1 expression over time in wild-type or *Tnf*^−/−^ BMDMs in response to 100 nM CpG (scaled to receptor levels before treatment). Error bars show standard deviations across three independently performed experiments, and double asterisks indicate a p value <0.001 using a Student’s t test comparing wild-type and *Tnf*^−/−^ levels at a particular timepoint. (D) Single-cell heatmaps of NFκB activation in RelA^V/V^ BMDMs in response to 100 nM CpG, with or without feedforward TNF signaling blocked using saturating amounts (5 mg/mL) of soluble TNFR2 co-injected with treatment. (E) Proportions of NFκB dynamic subtypes (off, transient, oscillatory, or persistent) as quantified from the data in (D). (F) Schematic depicting two cells. One cell (left) responds to CpG by activating NFκB and producing TNF that may act upon it in an autocrine manner. Another cell (right) does not respond to CpG (possibly because of low TLR9 expression), but responds to paracrine TNF and hence produces oscillatory NFκB activity.

To address this discrepancy, we noticed that within the population of diverse responses to CpG, oscillatory trajectories were generally slightly delayed compared to transient and non-oscillatory trajectories (Figure 7A). We therefore wondered whether cytokine feedback, especially by TNF (Caldwell et al., 2014), not represented in the simple mathematical models might be responsible for this discrepancy between model simulations and experimental observations. Indeed, we found that a small but statistically significant amount of TNF was detectable in the cell culture medium at the early 5-min time point of CpG stimulation (Figure 7B). Furthermore, flow cytometry for the TNF receptor revealed a rapid internalization of TNFR1 not only in response to TNF but also CpG, which was also TNF-dependent (Figure 7C). To test whether paracrine TNF signaling was in fact responsible for oscillatory NFκB responses, we measured single-cell dynamic responses to CpG in the presence or absence of saturating levels of recombinant soluble TNFR2 (Figure 7D). We noted a substantial decrease in oscillatory trajectories, and the fraction of non-responding cells increased in the TNF blocking condition (Figures 7D and 7E). Similar observations were made with LPS (Figures S7H and S7I). Our data suggest that TNF produces oscillatory NFκB activity within cell populations exposed to low levels of CpG. We imagine that cells, which are unresponsive to CpG due to, for example, low TLR9 levels, may still respond to TNF produced by cells in the population that are responsive to CpG, possibly because of higher levels of TLR9 (Figure 7F). Thus, in the context of MyD88-mediated PAMPs, oscillatory NFκB may be an indicator of paracrine signaling by host factor TNF.

## DISCUSSION

In this work, we report the identification of six dynamical features that characterize complex, stimulus-specific time-course trajectories of NFκB activities in single primary macrophage cells. Using information-theoretic and machine learning approaches, we show that these function as codewords (termed “signaling codons”) to convey information about the extracellular environment to nuclear target genes. In an inflammatory disease mouse model, diminished ligand-specific deployment of two signaling codons—“oscillation” and “duration”—results in greater confusion of ligand sensing and diminished stimulus specificity in gene expression that may contribute to the pathology. Our investigation of the molecular mechanisms underlying the stimulus-specific generation of NFκB signaling codons revealed simple circuit motifs responsible for each; and it revealed that NFκB oscillations observed in macrophages are in fact often a hallmark of paracrine TNF.

These findings were made possible by our development of experimental and computational tools that provided an unprecedented quantity and quality of experimental data of NFκB in primary cells responding to diverse immune threats. As cell lines show reduced responsiveness (Cheng et al., 2015), and ectopic expression of reporters can lead to artifactual oscillatory dynamics (Barken et al., 2005), we generated the NFκB RelA^V/V^ mouse strain allowing us to image primary macrophages, the cell type that functions as the sentinels of the immune system. We were able to study the specificity of NFκB responses to many stimulus conditions encompassing multiple doses of pathogen-derived and host-derived ligands to which these primary macrophages respond vigorously. A robust automated image analysis pipeline, and described information-theoretic analysis and machine learning classification workflows, enabled a rigorous, quantitative analysis of over 4.9 million single-cell data points derived from 44 distinct time-course conditions, not including biological replicates.

To identify signaling codons, i.e., informative dynamical features, we employed an information theoretic framework. Previous applications of an information theoretic framework related the timeseries of nuclear NFκB abundance at either one or several time points to different doses of stimulus (Cheong et al., 2011; Selimkhanov et al., 2014). While it was shown that time course measurements can provide more information about ligand and dose than a single time point (Selimkhanov et al., 2014), it remained unclear which dynamical features are important in conveying this information. Prior studies sought to characterize temporal NFκB trajectories in terms of *ad hoc*-defined dynamical features such as duration (Hoffmann et al., 2002; Werner et al., 2005) or “inter-peak time/frequency” (Hughey et al., 2015). However, these features were not tested for information content, though some appear to correlate with gene expression responses (Lane et al., 2017; Martin et al., 2020). Our datasets and analytical workflow allowed for an unbiased evaluation of hundreds of potential features and yielded six that essentially define stimulus-specific NFκB dynamics for the five ligands at multiple doses tested here. As these dynamic signaling features optimally provide the nucleus with information about the extracellular environment, they are codewords of a signaling code. We showed that signaling codons identified by the information-theoretic approach are sufficient for a machine to learn to correctly classify NFκB trajectories in terms of stimulus and dose. Interestingly, inter-peak time or “period” were not represented (i.e., frequency is not stimulus specific), but instead, the presence or absence of oscillatory content emerged as an important signaling codon—it is key to distinguishing PAMP-responsive and cytokine TNF-responsive NFκB dynamics. It will be of interest if additional datasets from macrophages or other cell types will yield additional codewords of the NFκB signaling code.

We have begun to characterize the key mechanisms that encode the six signaling codons of the NFκB signaling code. Building upon prior mathematical models that have investigated NFκB dynamics in response to a single ligand in immortalized cell lines (Basak et al., 2012), the model presented here recapitulates both oscillatory and non-oscillatory trajectories in primary macrophages in response to five ligands at several different doses and provides insights into the molecular mechanisms. As the IκB-NFκB signaling module is common to all stimulus-response pathways, and the IκBα negative feedback loop indeed supports both oscillatory and non-oscillatory activities (our finding), stimulus-specific deployment of the six signaling codons depends on the biochemical characteristics of components in the receptor-associated signaling modules. Key characteristics are (1) the ligand half-life, as short half-lives (e.g., TNF) render the duration of the response dependent on stimulus concentration (Barken et al., 2005; Cheong et al., 2006); (2) the receptor translocation and replenishment rates that may either allow for post-stimulation shutdown or second phase signaling (Becker et al., 2010); (3) the dose response of the adaptor (TRAFs, MyD88, TRIF), as, for example, oligomerized MyD88 tends to digitize responses, but TRIF does not (Cheng et al., 2015); and (4) the deactivation kinetics of adaptors and ubiquitin chain networks that are likely key determinants of the termination of signaling but require further biochemical characterization. While the present model qualitatively recapitulates representative NFκB trajectories for each stimulus, developing a model that quantitatively recapitulates the heterogenous population response will require innovations in parameter fitting such a large model and in developing an objective function that captures biological meaningful information of each stimulus response.

It is well established that the temporal trajectories of NFκB activity are correlated with gene expression (Gutschow et al., 2019; Hoffmann et al., 2002). Prior work has described molecular mechanisms that particular target genes employ to “decode” specific NFκB signaling codons. “Peak amplitude/fold change” for example, was described to be sensed effectively by an incoherent feedforward loop involving the NFκB-responsive generation of p50 homodimers (Lee et al., 2014a). Stimulus-specific duration was found to be differentiated by two mechanisms; whereas stimulus-specific expression of core regulators of the inflammatory response was mediated by an mRNA half-life of a few hours, pro-inflammatory initiators tend to employ a chromatin-based mechanism that involves the movement of a nucleosome (Sen et al., 2020). However, the oscillatory/non-oscillatory codon does not seem to control the stimulus-specific expression of NFκB primary response target genes (Barken et al., 2005). Our scRNA-seq data indicate that the stimulus-specific deployment of the oscillatory codon is critical to ensuring the stimulus-specific activation of the IRF/IFN pathway. This is an important insight that may explain the connection between inflammatory dysregulation of NFκB and the interferon dysregulation associated with autoimmune disease (Marketos et al., 2019; Muskardin and Niewold, 2018). However, the mechanism by which the “oscillatory” codon is decoded by immune response genes requires further study. Because immune response genes are regulated by multiple transcription factors, the misregulation of one may not result in misregulated immune response gene expression.

A hallmark of all single-cell datasets is the heterogeneity within an isogenic, identically stimulated population. Hence, it is not surprising that the stimulus specificity of the dynamical features identified here is by no means perfect, and that a machine learning classifier applied to all features or the six most informative signaling codons revealed some confusion, particularly among the NFκB responses to three bacterial PAMPs. Confusion here means, for example, that some (but not all) cells stimulated with CpG produce NFκB responses that are indistinguishable from some (but not all) cells stimulated with Pam3CSK4. We suggest that the capacity (or lack thereof; i.e., confusion) for mounting specific responses is a fundamental, functional characteristic of macrophages as immune sentinel cells. Furthermore, given a macrophage’s functional plasticity, we expect that this capacity for stimulus discrimination be similarly tuned—determined by the context of microen-vironmental cytokines and exposure histories. In this study, macrophages derived from a mouse model of the systemic inflammatory disease, Sjögren’s syndrome, showed increased levels of ligand confusion. This particular model involves genetic variants in the IκBα promoter, but the impact on NFκB signaling dynamics at the single-cell level was unknown. While cells are capable of responding to diverse immune threats, the reduction in specificity adds to our understanding of this systemic autoimmune disease and may contribute to its etiology. Future studies will address whether other autoimmune or inflammatory diseases may in fact be triggered by a diminished response specificity or increased confusion to diverse immune stimuli.

### Limitations of study

The present study identifies six informative dynamical features (signaling codons) within diverse temporal NFκB activation dynamics in macrophages. It is likely that in other stimulus conditions or cell types, or when studying other signal transducers, other dynamical features may be identified that are critical for accurate classification of immune threats. Thus, signaling codes are not as universal and uniformly precise as the genetic code, but context dependent, evolving, and subject to imprecision, as oral language. While we show that the stimulus-specific deployment of two signaling codons is defective in macrophages derived from a Sjögren’s mouse model, we have not shown whether or how that defect causally relates to the reported loss in stimulus-specific gene expression. Furthermore, whether or how those molecular-level observations causally relate to the pathology of Sjögren’s syndrome in humans requires further study—the current work merely motivates the articulation of a hypothesis: that the etiology of some inflammatory diseases may be signal confusion based on defective signaling codon deployment.

## STAR★METHODS

### RESOURCE AVAILABILITY

#### Lead contact

Further information and requests for resources and reagents should be directed to and will be fulfilled by the Lead Contact, Alexander Hoffmann (ahoffmann@ucla.edu)

#### Materials availability

Mouse lines generated in this study are available upon request.

#### Data and code availability

All data are available at https://data.mendeley.com/datasets/6wksmvh5p4/draft?a=832656ba-2bde-40a4-8bbc-4cecb1d9543d. Software for image processing available at https://github.com/brookstaylorjr/MACKtrack. Software for computational simulations of NFκB dynamics is available at https://github.com/Adewunmi91/nfkb_model.

### EXPERIMENTAL MODEL AND SUBJECT DETAILS

#### Mouse models

The mVenus-RelA (RelA^V/V^) endogenously-tagged mouse line was generated by Ingenious Targeting Laboratory. A donor sequence encoding the monomeric variant of the Venus fluorescent protein (Koushik et al., 2006) joined by a short flexible linker sequence directly upstream of the start codon of the murine *Rela* locus was used to generate, via homologous recombination, a tagged embryonic stem cell line, that was implanted to yield heterozygous mice. These mice were then bred with a mouse line constitutively expressing the *Flp* recombinase to remove the *Neo* resistance marker included in the homologous donor sequence. We then back-crossed the resultant mice with wild-type C57BL/6J mice to remove the *Flp* background and generate homozygously tagged mice (RelA^V/V^). mVenus-RelA mice were crossed into a IκBα^−/−^TNF^−/+^cRel^+/−^ line (TNF and cRel heterozygosity are required to rescue embryonic lethality of the IκBα^−/−^ genotype) (Shih et al., 2009), as well as into an IκBβ^−/−^ IκBε^−/−^ line (Hoffmann et al., 2002). For the Sjӧgren’s syndrome mouse model, we crossed mVenus-RelA mice into a strain that harbors mutated κB sites in the IκBα promoter (Peng et al., 2010).

#### Macrophage cell culture

Bone marrow-derived macrophages (BMDMs) were prepared by culturing bone marrow monocytes from femurs of 8–12 week old mice in CMG 14-12-conditioned medium using standard methods (Cheng et al., 2015; Takeshita et al., 2000). BMDMs were re-plated in experimental dishes on day 4, then were stimulated on day 7. BMDMs were stimulated with indicated concentrations of lipopolysaccharide (LPS, Sigma Aldrich), murine TNF (R&D), a TLR1/2 agonist, the synthetic triacylated lipoprotein Pam3CSK4 (PAM), a TLR3 agonist, low molecular weight polyinosine-polycytidylic acid (poly(I:C) (PIC)), a TLR9 agonist, the synthetic CpG ODN 1668 (CpG).

### METHOD DETAILS

#### Biochemical assays

For immunoblots of whole cell lysates, bone-marrow derived macrophages were replated on day 4 at 20,000/cm^2^ in 6-cm dishes or 6-well plates. After stimulation on day 7, sample buffer was added directly after washing cells with PBS. Immunoblots followed standard procedure with anti-RelA (sc-372, Santa Cruz Biotechnology), anti-pIKK (CST2697), and anti-IKK2 (CST2678). Western blot band intensities were quantified using ImageJ. Nuclear extract preparation and electrophoretic mobility shift assays followed published procedures (Caldwell et al., 2014).

#### Live-cell imaging

Bone-marrow macrophages were replated on day 4 at 20,000 or 15,000/cm^2^ in an 8-well ibidi SlideTek chamber, for imaging at an appropriate density (approx. 60,000/cm^2^) on day 6 or day 7. 2 h prior to stimulation, cells were incubated for 5 min at room temperature in a solution of 2.5 ng/mL Hoechst 33342 in PBS, then BMDM culture media was replaced. This staining condition was optimized to ensure no loss of cell viability and no aberrant morphological changes over a 24 h period of imaging in the conditions described below. Cells were imaged at 5-min intervals on a Zeiss Axio Observer platform with live-cell incubation, using epifluorescent excitation from a Sutter Lambda XL light source. Images were recorded on a Hamamatsu Orca Flash 2.0 CCD camera. After the start of imaging, additional culture media containing stimulus (TNF, LPS, poly(I:C), CpG, or Pam3CSK4) was injected into the chamber *in situ*. We have documented the reliability of the imaging workflow by establishing that distinct biological replicates give reproducible data (Figure S1D) and that distinct imaging frames of the same well provide reproducible data (Figure S1E). All data are available at https://data.mendeley.com/datasets/6wksmvh5p4/draft?a=832656ba-2bde-40a4-8bbc-4cecb1d9543d.

#### Measurement of TNF secretion and surface TNF receptor expression

To measure TNF secretion, bone-marrow macrophages were replated on day 4 at 25,000/cm^2^ in a 96-well format. On day 6, media was refreshed with 80 μL media containing indicated treatment (TNF, LPS, or CpG). Supernatants were collected from wells, in triplicate, at indicated time points, using procedures from the murine TNF alpha ELISA Ready-SET-Go! kit (eBioscience #88-7324-88). To optimize assay sensitivity, measurement was performed in a half-area 96-well plate (Corning #3690), and sample incubation was performed overnight at 4°C. Fluorescence measurements were performed using a standard spectrophotometer.

To measure surface receptor expression, bone-marrow-derived macrophages were replated on day 4 at 20,000/cm^2^ in 6-cm dishes. On day 6, media was refreshed with 3 mL media containing indicated treatment (TNF, LPS, or CpG). At indicated time point, media was rinsed out with cold PBS. Cells were incubated with fluorophore-conjugated antibodies for TNFR1, CD11b, and F4/80 (Bio-Legend #113005, eBioscience #11-0112-82, eBioscience #12-4801-82) and analyzed, in triplicate by flow cytometry. Antibody concentration and staining conditions were performed according to manufacturer recommendations. Stained cells were measured using an Accuri C6 Flow Cytometer (BD Biosystems). Fluorescence compensation and live/dead cell filtering was performed in FlowJo v10.

#### Measurement of single cell RNA-seq expression

BMDMs were generated from 12-week-old WT and Sjögren’s Syndrome mice, re-plated in experimental dishes on day 5 of differentiation, and stimulated on day 7 for 8 h with 100 ng/mL lipopolysaccharide (LPS, Sigma Aldrich), 10 ng/mL murine TNF (R&D), and 50 μg/mL low molecular weight polyinosine-polycytidylic acid (poly(I:C)), or media only (Untreated control). Cells were then lifted into suspension by incubating at 37 C for 5 min using Accutase, labeled with TotalSeqB hashtag antibodies (TotalSeq-B0305 – B0308 anti-mouse Hashtag Antibody) and pooled, and captured using the 10x single cell sequencing protocol. Cell viability was ensured to be > 90% at the time of capture. Libraries were prepared with the Chromium Single Cell 3′ GEM Kit, Version 3.1 Chemistry. Hashtag libraries made using the Chromium Single Cell 3′ Feature Barcode Library Kit. Samples were sequenced paired-end 2×50 on an Illumina NovaSeq 6000 instrument.

### QUANTIFICATION AND STATISTICAL ANALYSIS

#### Image analysis

Microscopy time-lapse images were exported for single-cell tracking and measurement in MATLAB R2016a. The tracking routines followed those used in earlier work (Selimkhanov et al., 2014). Briefly, cells were identified using DIC images, then segmented, guided by markers from the Hoechst image. Segmented cells were linked into trajectories across successive images, then nuclear and cytoplasmic boundaries were saved and used to define measurement regions in other fluorescent channels, including mVenus-NFκB. Nuclear NFκB levels were quantified on a per-cell basis, normalized to image background levels, then were baseline-subtracted. Mitotic cells, as well as cells that drifted out of the field of view, were excluded from analysis. The toolboxes used for this analysis are available at https://github.com/brookstaylorjr/MACKtrack.

#### Channel capacity calculation and signaling codon identification

As there are ~9.3 × 10^16^ seven-dimensional combinations of 918 features (Table S3) and each channel capacity calculation takes ~90 s per combination, evaluating channel capacity of all combinations of features would take ~2.3 × 10^15^ h (~2.7 × 10^11^ years) to compute and is therefore is computationally infeasible. To narrow the search space, we utilized a feature selection approach. Since the channel capacities of individual features combine nonlinearly, there is no guarantee a high-ranking feature in low dimensional space will also be a subset of a high-ranking feature vector in high-dimensional space. Consequently, we utilized a forward feature selection approach that balances channel capacity rankings in lower dimensional space and diversity of candidates. Channel capacity calculations are performed on single dimensional features, ranked, and a subset of features above a threshold are selected to maximize diversity. As such 1D candidates are combined to form a set of 2D feature vectors. Channel capacity calculations are calculated on the 2D feature vectors, ranked and selected as in the 1D case. This iterative ranking and selection processes are repeated until additional dimensions offer no gain in channel capacity (Table S4).

Algorithmic detailed: We used Shannon’s information theoretic framework to correlate the stimulus condition to dynamical features extracted from temporal trajectories of NFκB activity.

$$\begin{array}{c} \text{noise}\\ ↓\\ X\to \text{communication channel}\to Y \end{array}$$

X= stimulus condition

Y=NFκB dynamical features

C(Q)=I(Y ; X)

$$I(Y;X)=H_{\text{diff }}(Y)-H_{\text{diff }}(Y|X)$$

$$H_{\text{diff }}(X)=\sum_{i=1}^{m}q_{i}H_{\text{diff }\left(X=x_{i}\right)}=-\sum_{i=1}^{m}q_{i}\sum_{j=1}^{n_{i}}\frac{1}{n_{i}}log2\left(f\left(X=x_{i}\right)\right)$$

$$H_{\text{diff }}(Y)=-\sum_{i=1}^{m}\frac{q_{i}}{n_{i}}\sum_{j=1}^{n_{i}}{\text{log}}_{2}\left(f\left(Y=y_{i_{j}}\right)\right)$$

$$f(Y=y)=\sum_{w=1}^{m}q_{w}f\left(Y=y|X=x_{w}\right)$$

$$H_{\text{diff }}(A)=-\sum_{j=1}^{N_{a}}\delta_{j}{\text{log}}_{2}\left(f\left(a_{j}\right)\right),\text{ where }\delta_{j}=\text{ probability of observing }a_{j}$$

$$f(A)=\frac{k}{N_{a}V_{d}z(A))\frac{d}{k}}$$

$$V_{d}=\frac{\pi^{\frac{2}{\sigma }}}{\Gamma \left(\frac{d}{2}+1\right)}$$

$$H_{\text{diff }}(Y|X)=\text{ conditional entropy}$$

m= number of stimulus conditions

n= number of cells in a condition

$$q_{i}=\text{ probability of observing a stimulus}$$

$$x_{ij}=a\text{ single cell}\prime \text{s response}$$

k= number of neighbors used in kNN estimate of marginal distribution of Y

d= vector dimension

$$\delta_{j}=\text{ probability of observation}$$

##### Controlling for different sample sizes

Jackknife resampling was used to control for different sample sizes by calculating channel capacity for differently-sized subsets and extrapolating to an infinite sample size.

$$n_{c}\text{=24}$$

##### Setting threshold

1. $t\leftarrow {\left(\frac{1}{\sqrt{2}}\right)}^{\left[1:6\right]}-{\left(\frac{1}{2}\right)}^{\left[1:6\right]}$
2. *t*_1_←0.3
3. If *d* > 6 then *t*←[*t*,0.1*1_*d*−6_]

Fori=1…d

1. Compute channel capacity by optimizing over marginal distribution of **X**
  - For *j* = 1…*k*
    - *c*_*j*_←*I*(*x*_*j*_; *Y*)
    - *q*_*j*_←*argmax*_*PX*_*I*(*x*_*j*_; *Y*)
2. Select a subset of feature vectors whose channel capacity values exceeds *t*_*i*_
  - X*←{*x*_*j*_|_*cj*_ > *t*_*i*_}}
  - Q*: = *argmax*_*PX*_*I*(X*; *Y*)
3. Select a subset of feature vectors that maximizes diversity of marginal distributions
  - Select feature vector that yields the maximum channel capacity
    $$\hat{x}\leftarrow \left\{x_{j}^{*}|c_{j}=\text{max}(c)\right\},\text{ equivalently }\hat{x}\leftarrow {\text{argmax}}_{x^{*}}I(X;Y)$$
  - Construct a set of feature vectors containing the $\hat{x}$ and feature vectors whose marginal distributions, *q*_*j*_, are most orthogonal to $\hat{q}\leftarrow {\text{argmax}}_{PX}I(\hat{x};Y)$
  - $x_{1}^{o}\leftarrow \hat{x}$, $q_{1}^{o}\leftarrow \hat{q}$
    - *Form* = 2…*n*_*c*_

1. $Q^{c}:=\left\{q|q\in Q^{*}\;\hat{q}\notin Q^{0}\right\}$
2. $q_{m}^{o}\leftarrow {\text{argmin}}_{Q^{c}}{\left\|Q^{{}^{\circ}}-Q^{c}\right\|}_{2}$
3. $x_{m}^{o}\leftarrow \left\{x_{j}^{*}|q_{j}^{*}\equiv q_{m}^{o}\right\}$

#### Machine learning classification

##### Construction of classification models

We trained an ensemble of 100 decision trees using the *fitcensemble* function from the Statistics and Machine Learning Toolbox from MathWorks. Decision tree models are simple, highly interpretable, and can be displayed graphically (James et al., 2013). Consequently, the decision process of the classifier can be easily interrogated. However, decision tree models have two key disadvantages: (1) mediocre prediction performance (Caruana and Niculescu-Mizil, 2006) and (2) high variance due to overfitting (James et al., 2013). Both disadvantages can be mitigated by aggregating an ensemble of decision trees. Empirical comparison of classification models shows that ensembles of decision trees outperform other classification algorithms across a variety of problem sets (Caruana and Niculescu-Mizil, 2006).

We used a bootstrap aggregation (bag) method for constructing the ensembles. Each tree in the ensemble is trained on a boot-strapped replica of the data—each replica is a random selection of the data with replacement. The predictions from the ensemble model are determined by a majority vote from each individual tree prediction. We trained the ensemble to learn the stimulus labels (TNF, Pam3CSK4, CpG, LPS, and poly(I:C)) from either the entire set of predictors (all 918 metrics, Table S6A) or a subset of predictors termed “signaling codons” (Table S6B).

##### Decision tree parameters

To construct each decision tree, the software considers all possible ways to split the data into two nodes based on the values of every predictor. Then, it chooses the best splitting decision based on constraints imposed by training parameters, such as the minimum number of observations that must be present in a child node (*MinLeafSize*) and a predictor selection criterion. The software recursively splits each child node until a stopping criterion is reached. The stopping criteria include (1) obtaining a pure node that contains only observations from a single class, (2) reaching the minimum number of observations for a parent node (*MinParentSize*), (3) reaching a split that would produce a child node with fewer observations than *MinLeafSize*, and (4) reaching the maximum number of splits (*MaxNumSplits*). We used default values for *MinLeafSize, MinParentSize*, and *MaxNumSplits*: 1, 10, sample size − 1, respectively (MathWorks, 2017). Loadings for classification models are listed in Table S6.

Since the standard prediction selection process at each node may be biased, we used a predictor selection technique, interaction-curvature test, which minimizes predictor selection bias, enhances interpretation of the model, and facilitates inference of predictor importance. The interaction-curvature technique selects a predictor to split at each node based on the *p-value*s of curvature and interaction tests. Whereas the curvature test examines the null hypothesis that the predictor and response variables are unassociated, the interaction test examines the null hypothesis that a pair of predictor variables and the response variable are unassociated. A node with no tests that yield *p-value*s ≤ 0.05 is not split. At each node, the predictor or pair of predictors that yield the minimum significant *p-value* (0.05) is chosen for splitting. To split the node, the software chooses the splitting rule that maximizes the impurity gain—difference in the impurity of the node (calculated using Gini’s diversity index) and the impurity of its children nodes (MathWorks, 2017).

##### Evaluation

We evaluated the performance of the classifiers using 5-fold cross-validation, or out-of-bag (OoB) validation, or an independent testing dataset. The OoB validation is virtually identical to K-fold cross-validation (Hastie et al., 2001) and imposes minimal computation costs. K-fold cross-validation increases the computational time by K fold. OoB is defined for bagged ensembles of decision trees (Hastie et al., 2001); whereas K-fold cross-validation can be used agnostic of the classification algorithm and is ubiquitous. We used OoB validation primarily to evaluate dose prediction models, which can be computationally impractical when the number features get large and K-fold cross-validation is used. We used the following performance metrics: true positive rate (recall), positive predictive value (precision), area under the Receiver Operating Characteristic (ROC) curve, F1 score, Matthews correlation coefficient, markedness, informedness and mean classification margin (Akosa, 2017; Powers, 2007; Vihinen, 2012).

##### Dose binary classification

A series of bagged decision trees were trained to classify no treatment controls and each stimulus (each dose of each ligand). The following hyperparameters were optimized using *fitcensemble* function in MATLAB: *NumLearningCycles*, *MinLeafSize*, *MinParentSize*, and *MaxNumSplits* were 33, 5, 2, and 100 respectively. The models were evaluated using 5-fold cross-validation. The performance metrics for the doses of each ligand were fitted to a polynomial curve using the *fit* function and *poly3* parameter.

##### Feature randomization

Features were selected at random to match the number of component features in codewords feature set (11) using the *randsample* function in MATLAB. The regenerator used was ml*fg6331_64*. The features were sampled 5 times. The performance values were averaged using arithmetic mean.

##### Feature autoencoding

We used a stacked autoencoder design with two autoencoders applied sequentially using *trainAutoencoder* and *encode* functions in MATLAB. The parameters for the first autoencoder are as follows: *MaxEpoch*, 400; *L2WeightRegularization*, 0.004; *SparsityRegularization*, 4; *SparsityProportion*, 0.15; *ScaleData*, false.

The parameters for the second autoencoder are as follows: *MaxEpoch*, 100; *L2WeightRegularization*, 0.002; *SparsityRegularization*, 4; *SparsityProportion*, 0.1; *ScaleData*, false.

#### Analysis of single cell RNA-seq data

Reads were aligned to mm10 using the 10x Cell Ranger software, version 4.0. Data was processed using Cell Ranger count to obtain a counts matrix. Data was filtered by removing cells with fewer than 1500 features. TotalSeqB hashtag labels were assigned to cells when > 75% of the cell’s hashtag reads came from one barcode. The *Seurat* R package (Stuart et al., 2019) was used to normalize the counts. PCA was run on scaled data, and Uniform Manifold Approximation and Projection (UMAP) was run through the *Seurat* R package using the top 20 principal components on WT and SS cells together.

To determine which genes had high stimulus-specificity, ANOVA was performed for each gene for only the three stimulus conditions in WT and SS. Estimation of maximum mutual information was performed using the R package *SLEMI* (Jetka et al., 2019). Machine learning was performed by training a random forest classifier, as implemented in the package *CARET* (Kuhn, 2008), on 70% of the WT data for the three stimulus conditions, using 10-fold cross-validation repeated three times, and with the mtry parameter set to sqrt(# of features). The metric used to evaluate the trained model was Accuracy, since the classes were relatively balanced. Differentially expressed genes displayed in heatmaps were found using Wilcoxon Mann Whitney U tests on each stimulus condition versus others, and the top 20 genes from each condition were merged for display. GSEA was run using the package *fastGSEA* (Korotkevich et al., 2019) on a list of genes ranked by the WT-SS difference in ANOVA F statistic, and motif analysis on the top 1000 ranked genes was done using HOMER (Heinz et al., 2010) against a whole genome background (Heinz et al., 2010; Korotkevich et al., 2019).

#### Mathematical modeling

##### Model structure

Several related models of NFκB activation in response to TNF have been established and iteratively parameterized (Ashall et al., 2009; Hoffmann et al., 2002; Tay et al., 2010), and used as a basis for modeling the NFκB response to LPS and other stimuli in immortalized cell lines with exogenously introduced (and overexpressed) fluorescent RelA (Cheng et al., 2015; Kellogg and Tay, 2015). The model presented here to account for NFκB dynamics in primary macrophages is closely based on these previous studies, inheriting identical model topologies where possible and minimizing any changes to parameter values.

##### Key experimental data constraints

As a first step toward parameterizing our model, we quantified characteristics of oscillatory endogenous BMDM signaling. We observed only slight differences in peak periodicity and amplitude between conditions (roughly a 10-min difference in median period for the lowest dose of TNF which induced robust oscillations, 0.33 ng/mL, and the highest dose tested). We did, however, observe pronounced differences in duration as the dose of TNF is increased (Figure S1F). Median period was determined to generally fall within 90–95 min, in the same range of oscillations measured in other cell types (Ashall et al., 2009; Tay et al., 2010).

The oscillatory frequency appeared to be remarkably stable across an extremely broad range of induction levels. Indeed, the variation observed across single cells in a particular condition (or even within the same cell) is much smaller than any differences in oscillations observed between conditions. Even when other stimuli are considered, the “signature” first harmonic of the oscillatory subpopulation remains consistent. This consistency across a wide range of input conditions agrees, notably, with predictions made using simplified discrete delay model of the NFκB network (Longo et al., 2013). These delays could plausibly arise from IkB mRNA (measured to be some 10–12 min) (Mor et al., 2010) and protein processing.

Biochemical assays indicate that the major difference between TNF and LPS-induced IKK activation is not in the maximum amplitude, but the duration of IKK induction (Shih et al., 2009; Werner et al., 2005). TNF strongly but transiently activates IKK. Peak IKK activity is limited in duration by rapid internalization and degradation of the ligand-bound receptor (Mosselmans et al., 1988; Watanabe et al., 1988; Werner et al., 2008). LPS-bound TLR4 is also rapidly internalized, but continues to strongly activate IKK from the endosome (Zanoni et al., 2011). This difference is reflected in single-cell NFκB activation: while the speed of NFκB activation (roughly proportional to the peak of IKK activity) is similar between TNF and LPS, sustained high levels of IKK activity in response to LPS leads to higher peak activity (Figures S5B and S5C).

##### Model fitting

The model was first fit for TNFR signaling and TLR4 signaling, as prior work established mathematical models that recapitulate population level data (Cheng et al., 2015; Werner et al., 2008). For the IKK-IκB-NFκB core module, model topology and parameters were confined to be near previously established values (Table S7). We performed a multidimensional sweep of transport rates and found a narrow range of parameters that could account for the observed frequency invariance, with high IKK activity diminishing oscillatory behavior (Figure S5D). Subsequent fitting to representative NFκB trajectories (using rmsd as distance metric) allowed us to optimize other parameters, including the induced synthesis rate constant of IκBα and the activation rate constant of IKK. For the receptor-associate modules, we required the model to recapitulate rapid IKK de- and re-activation (Behar et al., 2013), which allowed IKK responses to be both adaptive (in the case of TNF) and long duration (as in TLR4 responses). We employed a screen where repeated, random initialization of parameters (within an iteratively narrower range) was followed by their optimization via gradient descent (fmin function), fitting model simulations to representative NFκB trajectories. This two-stage sweep/fitting process was repeated until parameter values converged and fits to NFκB trajectory data could no longer be improved.

To parameterize the TLR1/2, TLR3, and TLR9 associated signaling modules, we used prior estimates of each receptor’s abundance in monocytes/macrophages (O’Mahony et al., 2008) to estimate synthesis and degradation rates. In many cases, receptor-ligand affinities were also known (Leonard et al., 2008; Nakata et al., 2006; Rutz et al., 2004) and were therefore used to estimate association and dissociation of the receptor. The kinetics of each receptor’s association with a downstream adaptor (TRIF or MyD88) were taken from estimates from our TLR4 model. NFκB responses to TLR9 were observed to be more transient than to either TLR4 or TLR1/2, in agreement with previous data (Caldwell et al., 2014) and the observed self-inactivation of TLR9 (Lee et al., 2014b).

The software to run the model is available at https://github.com/Adewunmi91/nfkb_model.

## Supplementary Material

1

2

3

4

5

6

7

8

9

10

## Figures and Tables

**Table:** KEY RESOURCES TABLE

REAGENT or RESOURCE	SOURCE	IDENTIFIER
Antibodies		
PE-conjugated Anti-Mouse F4/80 Antigen	eBioscience	Cat# 12-4801-82; RRID:AB_465923
FITC-conjugated Anti-Mouse CD11b	eBioscience	Cat# 11-0112-82; RRID:AB_464935
APC anti-mouse CD120a (TNF R Type I/p55)	BioLegend	Cat# 113005; RRID:AB_2208780
Anti-RelA Ab	Santa Cruz Biotechnology	Cat# sc-372; RRID:AB_632037
Anti-pIKK	CST	Cat# 2697; RRID:AB_2079382
Anti-IKK2	CST	Cat# 2678; RRID:AB_2122301
Chemicals, peptides, and recombinant proteins		
LPS	Sigma, B5:055	L2880
murine TNF	Roche	11271156001
Pam3CSK4	Invivogen	tlrl-pms
low MW polyinosine-polycytidylic acid (Poly(I:C))	Invivogen	tlrl-picw
synthetic CpG ODN 1668	Invivogen	tlrl-1668
Recombinant Mouse sTNFRII/TNFRSF1B	R & D Systems	426-R2–050
high MW polyinosine-polycytidylic acid (Poly(I:C))	Invivogen	tlrl-picw
Critical commercial assays		
Direct-zol RNA isolation kit	Zymo Research	R2060
TruSeq Stranded mRNA Library Prep Kit	Illumina	RS-122–2101
Mouse TNF alpha ELISA Ready-SET-Go! kit	eBioscience	#88-7324-88
TotalSeq^™^-B 0305 anti-mouse Hashtag Antibody	BioLegend	Cat# 155839; RRID:AB_2814071
TotalSeq^™^-B 0306 anti-mouse Hashtag Antibody	BioLegend	Cat# 155841; RRID:AB_2814072
TotalSeq^™^-B 0307 anti-mouse Hashtag Antibody	BioLegend	Cat# 155843; RRID:AB_2814073
TotalSeq^™^-B 0308 anti-mouse Hashtag Antibody	BioLegend	Cat# 155845; RRID:AB_2814074
Chromium Single Cell 3ʹ; GEM Version 3.1	10x Genomics	PN-1000121
Chromium Single Cell 3ʹ Feature Barcode Library Kit	10x Genomics	PN-1000079
Deposited data		
Single cell NFκB signaling dynamics	This paper	Mendeley Data: https://doi.org/10.17632/6wksmvh5p4.1
10x BMDM scRNaseq	This paper	GSE162992
Experimental models: Organisms/strains		
RelA^mVenus/mVenus^ (C57BL/6)	this paper	mVenus-RelA
mVenus-RelA^+/−^ IkBb^−/−^ IkBe^−/−^ (C57BL/6)	this paper	IkBb^−/−^, IkBe^−/−^
mVenus-RelA^+/−^ IkBa^−/−^ TNF^+/−^ cRel^+/−^ (C57BL/6)	this paper	IkBa^−/−^
mVenus-RelA^+/+^ IkBa ^M/M^ (C57BL/6)	this paper	Sjögren’s, SS
Software and algorithms		
MATLAB R2016a - Image processing, data analysis, and modeling	MathWorks	http://mathworks.com
MACKtrack - Cell tracking and single-cell measurement (MATLAB package)	This paper	https://github.com/brookstaylorjr/MACKtrack
nfkb_dynamics – dynamical feature computation	This paper	https://github.com/Adewunmi91/nfkb_dynamics
nfkb_model – multi-stimulus NFκB model	This paper	https://github.com/Adewunmi91/nfkb_model
Information_theory – channel capacity and mutual information computations	This paper	https://github.com/Adewunmi91/information_theory
FlowJ - Flow cytometry data processing	FlowJo, LLC	https://www.flowjo.com/
R - Statistical analysis	R Foundation	https://www.r-project.org/
Cell Ranger 4.0	10x Genomics	https://support.10xgenomics.com/single-cell-gene-expression/software/overview/welcome
CARET	(Kuhn, 2008)	http://caret.r-forge.r-project.org/
HOMER	(Heinz et al., 2010)	http://homer.ucsd.edu/homer/
Seurat	(Stuart et al., 2019)	https://www.rdocumentation.org/packages/Seurat/versions/3.1.4
SLEMI	(Jetka et al., 2019)	https://cran.r-project.org/web/packages/SLEMI/index.html
fastGSEA	(Korotkevich et al., 2019)	http://bioconductor.org/packages/release/bioc/html/fgsea.html

## Highlights

- Primary macrophages produce ligand- and dose-specific NFκB activation dynamics
- Six dynamical features, i.e., codons, inform the nucleus about the stimulus
- Oscillations inform about host-cytokine TNF, not MyD88-mediated immune threats
- Machine learning reveals substantial codon confusion in Sjögren’s macrophages
