# Supplementary material for: Six distinct NFκB signaling codons convey discrete information to distinguish stimuli and enable appropriate macrophage responses
Source: Immunity. Author manuscript; Available in PMC 2021 Jun 7. (PMC8184127; doi:10.1016/j.immuni.2021.04.011)
Supplement: 1 [file NIHMS1708596-supplement-1.pdf]

Immunity, Volume *54*

Supplemental information

Six distinct NFκB signaling codons convey discrete information to distinguish stimuli and enable appropriate macrophage responses

Adewunmi Adelaja, Brooks Taylor, Katherine M. Sheu, Yi Liu, Stefanie Luecke, and Alexander Hoffmann

This PDF file includes:

Figures S1 to S7. Related to Figures 1 to 7, respectively.

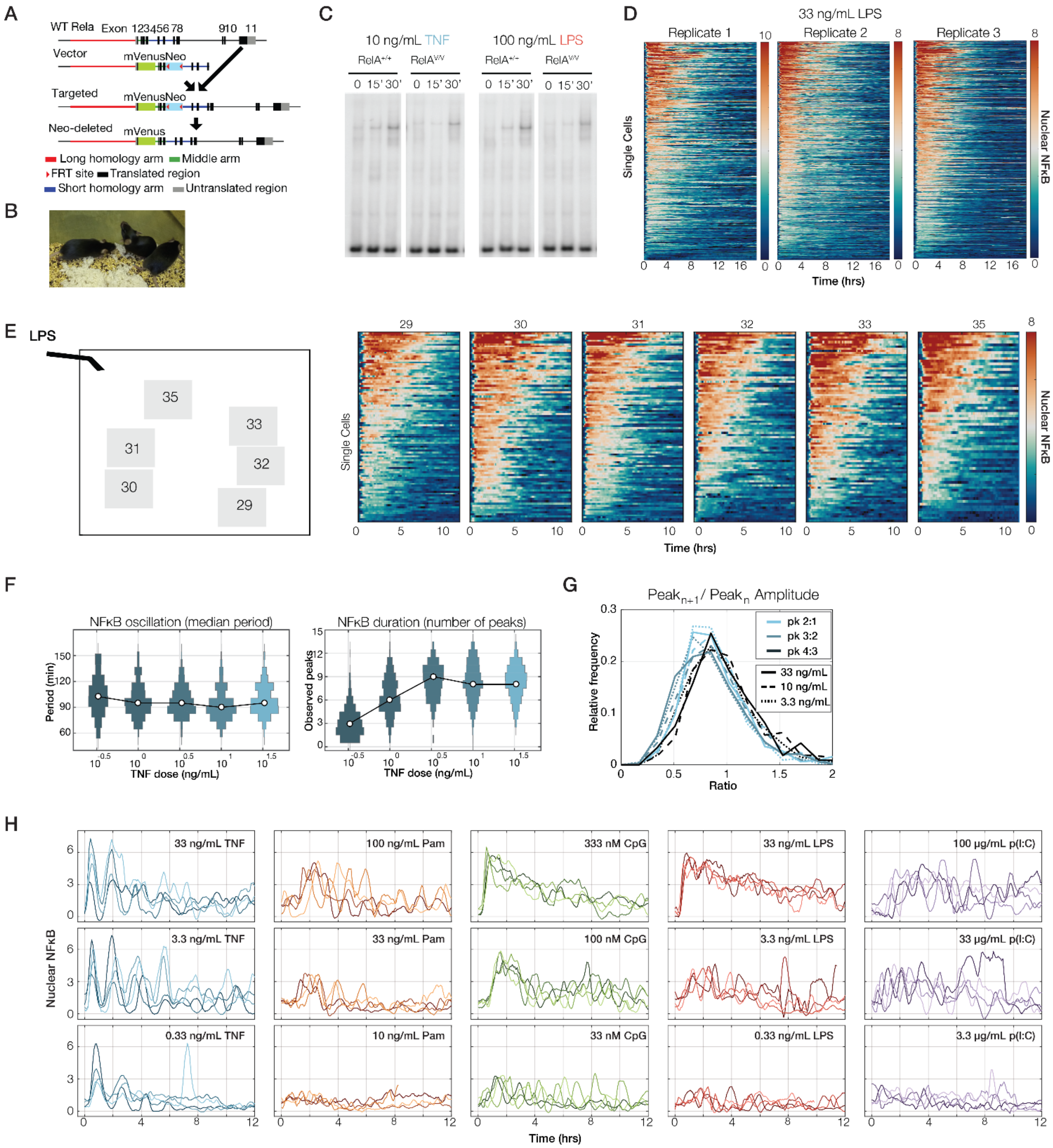

Figure S1. An experimental model and imaging workflow allows for reliable tracking NFκB RelA dynamics in primary macrophages at single cell level, Related to Figure 1.

(A) Schematic of the homologous recombination strategy for generating the mVenus-RelA allele in embryonic stem cells. These were injected into blastocysts for RelA$^{v/v}$ mouse generation.

(B) Image of homozygous RelA$^{v/v}$ mice shows that they are overtly healthy.

(C) mVenus-RelA macrophages show normal levels of NFκB DNA binding activity. NFκB EMSA of nuclear extracts made from mVenus-RelA and wild-type control BMDMs stimulated for 0, 15', and 30' with 10 ng/mL TNF and 100 ng/mL LPS.
(D) Experimental live cell imaging workflow and automated image analysis is robust as documented by biological replicates produced months apart from different mice.
(E) Microscopy workflow shows no location bias. Fields of view used in replicate 2 of 33 ng/mL LPS condition (left). Heatmaps of NFκB responses of cells in different fields of view (right).
(F) TNF dose does not regulate oscillation period but duration. Violin plots showing distributions of single-cell oscillation period (median peak-to-peak time) and duration (number of peaks measured in 18 hrs) across a range of TNF stimulus levels.
(G) TNF oscillations do not have a primary first peak, but rather steadily diminishing peaks. Histograms of oscillatory peak ratios (i.e. between amplitudes of subsequent peaks in the same cell) in response to multiple doses of TNF.
(H) Representative trajectories of nuclear mVenus-RelA localization in response to three doses of each ligand.

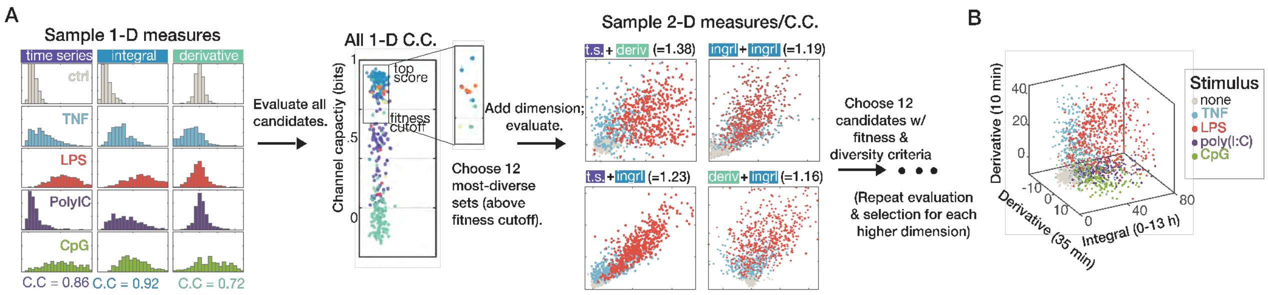

Figure S2. Design of an algorithm to identify information-maximizing combinations of dynamic features, Related to Figure 2.
(A) Procedure: single-dimension measurements (shown as histograms of cell population for each input condition) are evaluated across all input conditions. The output channel capacities are ranked: a subset of the candidates above a minimum "fitness" threshold are then selected to maximize diversity. These candidates are then re-evaluated in conjunction with a second dimension. This ranking/selection process is repeated until the final dimension is reached and a multidimensional vector is assembled.
(B) A sample representation of an optimal three-dimensional vector capturing single-cell measurements of NFκB responses. Using a three-dimensional vector, NFκB responses are quantified and each cell responding to the indicated ligand is depicted in a three-dimensional graph.

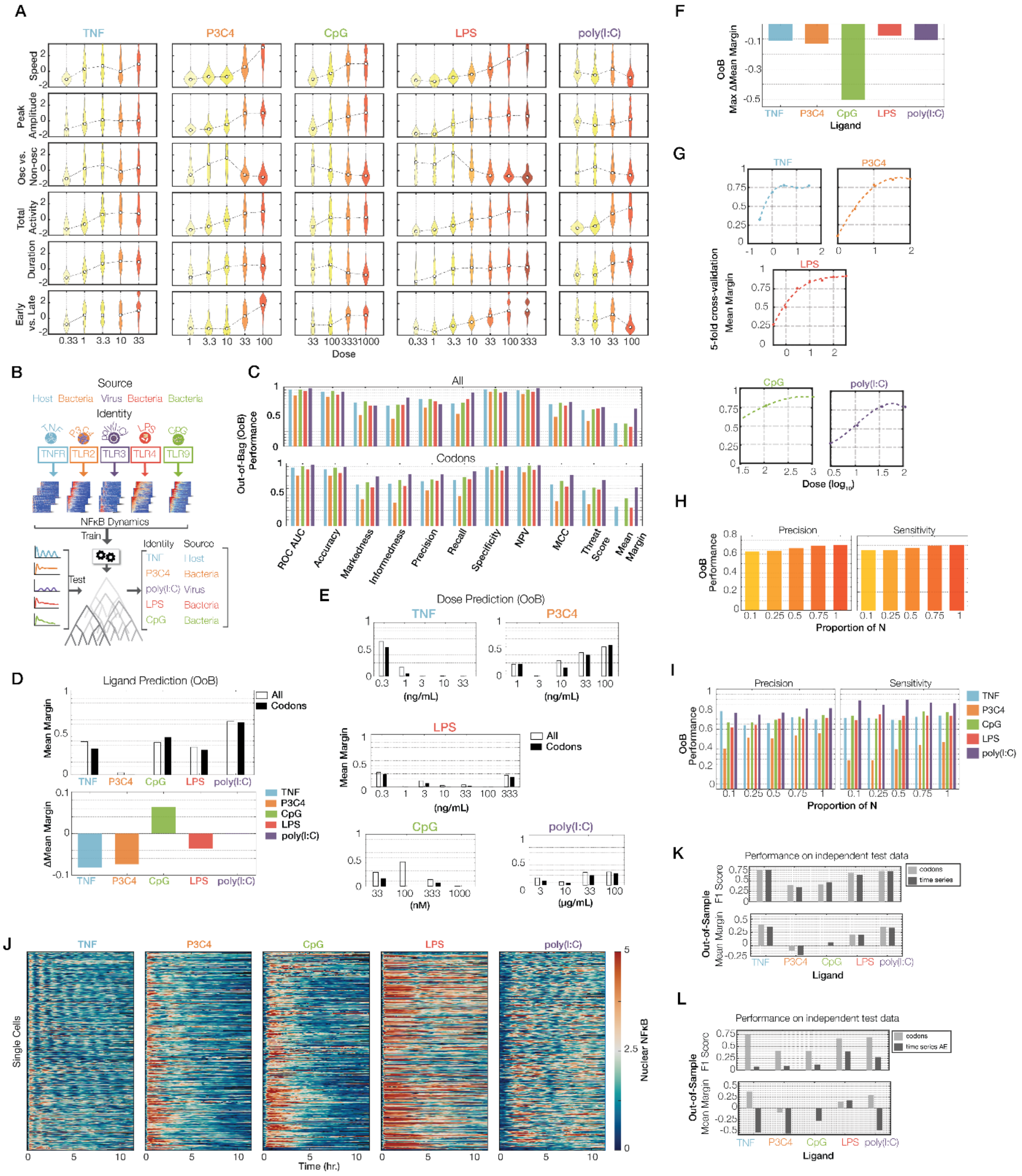

Figure S3. Evaluating the sufficiency and necessity of signaling codons, Related to Figure 4.

(A) Dose-dependent deployment of signaling codons. Violin plots of the relative presence of indicated signaling codons (z-score) in the trajectories of individual cells.

(B) Machine learning classification procedure: Predictors/features of NFκB signaling dynamics in response to TNF, Pam3CKS4, poly(I:C), LPS, and CpG were used to train an ensemble (using bootstrap aggregation) of 100 decision tree models to predict ligand identity and ligand source.

(C) Six signaling codons perform as well as all 918 dynamical features. A variety of metrics to ascertain ligand classification performance using all features (top) and using only signaling codons (bottom).

(D) Comparison of ligand classification margin (probability of the correct class minus the highest probability of the incorrect classes) of models trained using all predictors versus signaling codons: (top) mean classification margins across all ligands; (bottom) difference of mean classification margin of signaling codon classifier and all predictors classifier. Models validated using out-of-bag (OoB) observations.

(E) Comparison of dose classification margins of classifiers trained on all predictors versus signaling codons: mean classification margins across all doses for each ligand. Models validated using out-of-bag (OoB) observations.

(F) Maximum of the differences in mean classification margins between signaling codons classifiers and all predictor classifiers. Models validated using out-of-bag (OoB) observations.

(G) Dose dependence of ligand identification: mean margin of binary decision tree classifiers that distinguish no treatment controls from each ligand at the indicated dose. Models validated using 5-fold cross-validation.

(H) Classification performance as a function of training sample size averaged over ligands: steady increase in precision (left) and sensitivity (right) of classification as the number of training sample increases.

(I) Classification performance as a function of training sample size grouped by ligand: dependence of classification precision (left) and sensitivity (right) varies by ligand.

(J) Heatmaps of independent test data set.

(K) Comparison of classification performance using signaling codons versus time series using independent test (out-of-sample) data. F1 score (top) and mean margin (bottom) show similar performance.

(L) Comparison of classification performance using signaling codons versus autoencoder-transformed time series using independent test (out-of-sample) data. F1 score (top) and mean margin (bottom) show that ligand classification using autoencoder-transformed time series performance significantly worse on independent test data.

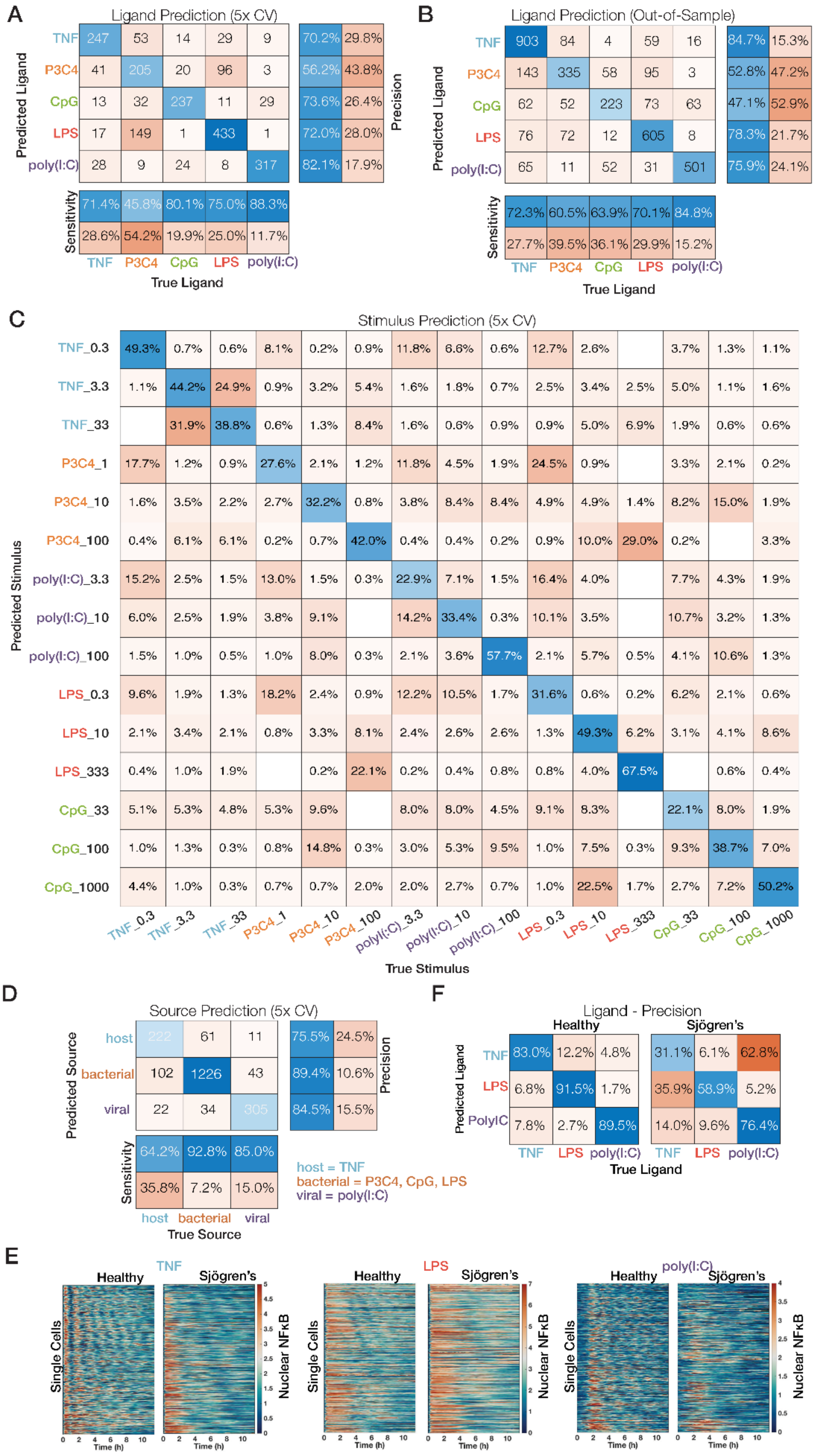
A
Ligand Prediction (5x CV)
Predicted Ligand
TNF 247 53 14 29 9 | 70.2% 29.8%
P3C4 41 205 20 96 3 | 56.2% 43.8%
CpG 13 32 237 11 29 | 73.6% 26.4%
LPS 17 149 1 433 1 | 72.0% 28.0%
poly(I:C) 28 9 24 8 317 | 82.1% 17.9%
Precision
Sensitivity
71.4% 45.8% 80.1% 75.0% 88.3%
28.6% 54.2% 19.9% 25.0% 11.7%
TNF P3C4 CpG LPS poly(I:C)
True Ligand
B
Ligand Prediction (Out-of-Sample)
Predicted Ligand
TNF 903 84 4 59 16 | 84.7% 15.3%
P3C4 143 335 58 95 3 | 52.8% 47.2%
CpG 62 52 223 73 63 | 47.1% 52.9%
LPS 76 72 12 605 8 | 78.3% 21.7%
poly(I:C) 65 11 52 31 501 | 75.9% 24.1%
Sensitivity
72.3% 60.5% 63.9% 70.1% 84.8%
27.7% 39.5% 36.1% 29.9% 15.2%
TNF P3C4 CpG LPS poly(I:C)
True Ligand
C
Stimulus Prediction (5x CV)
Predicted Stimulus
TNF_0.3 49.3% 0.7% 0.6% 8.1% 0.2% 0.9% 11.8% 6.6% 0.6% 12.7% 2.6% 3.7% 1.3% 1.1%
TNF_3.3 1.1% 44.2% 24.9% 0.9% 3.2% 5.4% 1.6% 1.8% 0.7% 2.5% 3.4% 2.5% 5.0% 1.1% 1.6%
TNF_33 31.9% 38.8% 0.6% 1.3% 8.4% 1.6% 0.6% 0.9% 0.9% 5.0% 6.9% 1.9% 0.6% 0.6%
P3C4_1 17.7% 1.2% 0.9% 27.6% 2.1% 1.2% 11.8% 4.5% 1.9% 24.5% 0.9% 3.3% 2.1% 0.2%
P3C4_10 1.6% 3.5% 2.2% 2.7% 32.2% 0.8% 3.8% 8.4% 8.4% 4.9% 4.9% 1.4% 8.2% 15.0% 1.9%
P3C4_100 0.4% 6.1% 6.1% 0.2% 0.7% 42.0% 0.4% 0.4% 0.2% 0.9% 10.0% 29.0% 0.2% 3.3%
poly(I:C)_3.3 15.2% 2.5% 1.5% 13.0% 1.5% 0.3% 22.9% 7.1% 1.5% 16.4% 4.0% 7.7% 4.3% 1.9%
poly(I:C)_10 6.0% 2.5% 1.9% 3.8% 9.1% 14.2% 33.4% 0.3% 10.1% 3.5% 10.7% 3.2% 1.3%
poly(I:C)_100 1.5% 1.0% 0.5% 1.0% 8.0% 0.3% 2.1% 3.6% 57.7% 2.1% 5.7% 0.5% 4.1% 10.6% 1.3%
LPS_0.3 9.6% 1.9% 1.3% 18.2% 2.4% 0.9% 12.2% 10.5% 1.7% 31.6% 0.6% 0.2% 6.2% 2.1% 0.6%
LPS_10 2.1% 3.4% 2.1% 0.8% 3.3% 8.1% 2.4% 2.6% 2.6% 1.3% 49.3% 6.2% 3.1% 4.1% 8.6%
LPS_333 0.4% 1.0% 1.9% 0.2% 22.1% 0.2% 0.4% 0.8% 0.8% 4.0% 67.5% 0.6% 0.4%
CpG_33 5.1% 5.3% 4.8% 5.3% 9.6% 8.0% 8.0% 4.5% 9.1% 8.3% 22.1% 8.0% 1.9%
CpG_100 1.0% 1.3% 0.3% 0.8% 14.8% 0.3% 3.0% 5.3% 9.5% 1.0% 7.5% 0.3% 9.3% 38.7% 7.0%
CpG_1000 4.4% 1.0% 0.3% 0.7% 0.7% 2.0% 2.0% 2.7% 0.7% 1.0% 22.5% 1.7% 2.7% 7.2% 50.2%
TNF_0.3 TNF_3.3 TNF_33 P3C4_1 P3C4_10 P3C4_100 polyI(:C)_3.3 poly(I:C)_10 poly(I:C)_100 LPS_0.3 LPS_10 LPS_333 CpG_33 CpG_100 CpG_1000
True Stimulus
D
Source Prediction (5x CV)
Predicted Source
host 222 61 11 | 75.5% 24.5%
bacterial 102 1226 43 | 89.4% 10.6%
viral 22 34 305 | 84.5% 15.5%
Precision
Sensitivity
64.2% 92.8% 85.0%
35.8% 7.2% 15.0%
host bacterial viral
True Source
host = TNF
bacterial = P3C4, CpG, LPS
viral = poly(I:C)
F
Ligand - Precision
Healthy
Sjögren's
Predicted Ligand
TNF 83.0% 12.2% 4.8% | 31.1% 6.1% 62.8%
LPS 6.8% 91.5% 1.7% | 35.9% 58.9% 5.2%
PolyIC 7.8% 2.7% 89.5% | 14.0% 9.6% 76.4%
TNF LPS poly(I:C) TNF LPS poly(I:C)
True Ligand
E
TNF
Healthy
Sjögren's
LPS
Healthy
Sjögren's
poly(I:C)
Healthy
Sjögren's
Single Cells
Nuclear NFκB
Time (h)

Figure S4. Analyzing stimulus confusion in macrophages from healthy and diseased mice, Related to Figure 4.

(A) Confusion matrix of ligand predictions using 5-fold cross-validation: diagonal values show correct predictions and off-diagonals values show incorrect predictions; (right) percentage of correct predictions (precision; in blue) and incorrect predictions (false discovery rate; in orange); (bottom) percentage of ligands correctly identified (sensitivity/recall; in blue) and not identified (miss rate/false negative rate; in orange).

(B) Same as (A) but using an independent test set.

(C) Precision confusion matrix of stimulus (3 doses per ligand) predictions using 5-fold cross-validation. TNF, LPS, and Pam3CSK4 doses in ng/mL; poly(I:C) doses in µg/mL; CpG doses in nM.

(D) Confusion matrix of ligand source predictions using 5-fold cross-validation.

(E) Heatmaps of nuclear NFκB in macrophages from healthy mice compared to Sjögren Syndrome mice in response to 10 ng/mL TNF, 100 ng/mL LPS, and 50 µg/mL poly(I:C).

(F) Confusion matrices for precision for the healthy and Sjögren Syndrome macrophage data (from Figure 4). Evaluated by 5-fold cross-validation and an independent test set.

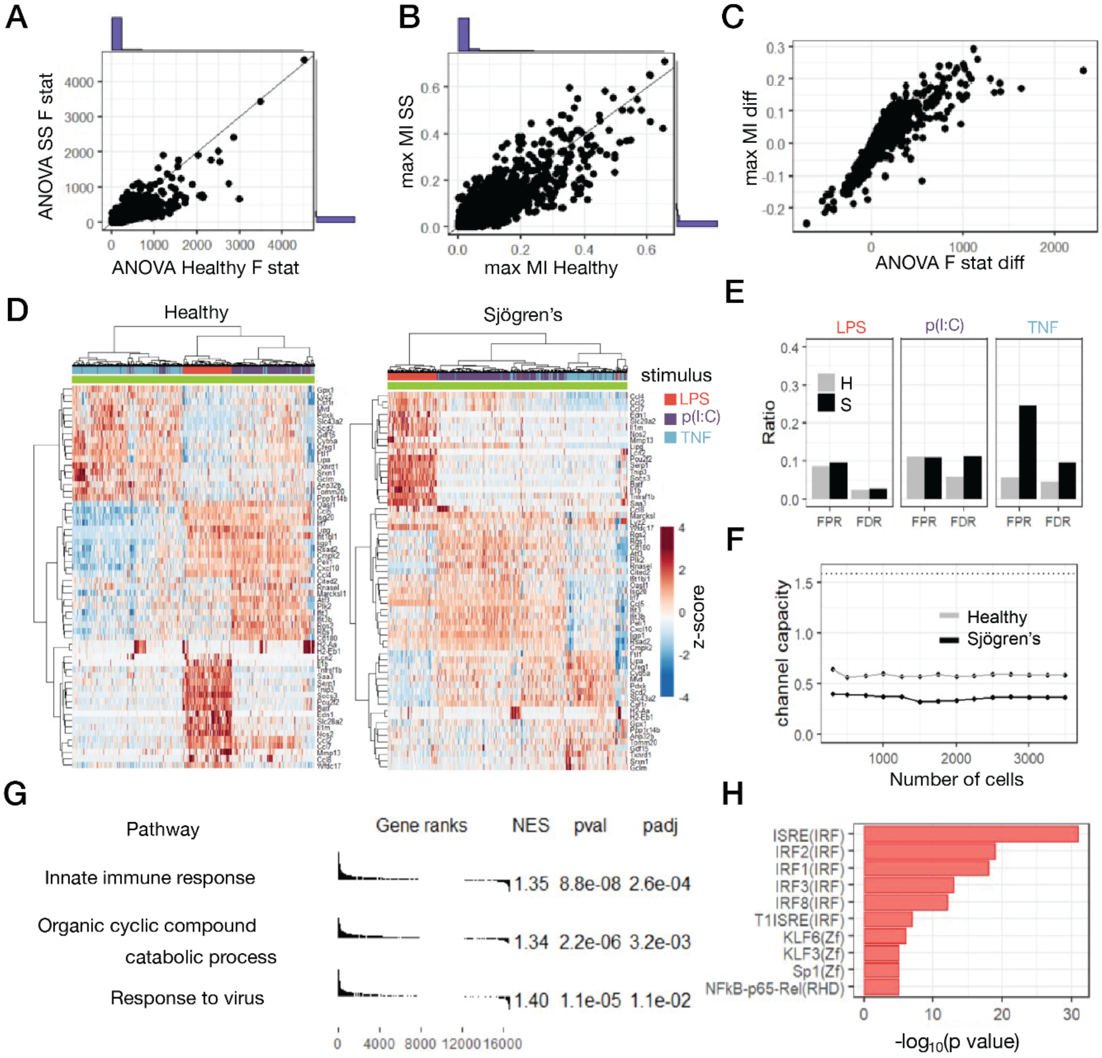

Figure S5. Single-cell gene expression in macrophages from healthy and Sjögren's Syndrome (SS) mice, Related to Figure 5.

(A) ANOVA F statistic comparing LPS, TNF, p(I:C) distributions for each gene in Healthy vs SS BMDMs.

(B) Maximum mutual information comparing LPS, TNF, p(I:C) distributions for each gene in Healthy vs SS BMDMs.

(C) Rankings from ANOVA F statistic difference and maximum MI difference between Healthy and SS are similar (Pearson's r = 0.83, p< 2.2e-16; Spearman's rho = 0.89, p<2.2e-16).

(D) Heatmap of z-scored gene expression of all stimulated cells in Healthy and SS mice, using top differentially expressed genes across the three stimulus conditions in Healthy.

(E) Dependence on cell numbers of channel capacity estimation for *Ccl5*.

(F) False positive and false discovery rates from random forest classifier for top 100 genes. (G) Top fGSEA results on mSigDB c5 Biological Processes collection for the full ANOVA ranked (Healthy- SS) gene list.

(H) Motif enrichment in the top 1000 genes ranked by ANOVA F statistic difference.

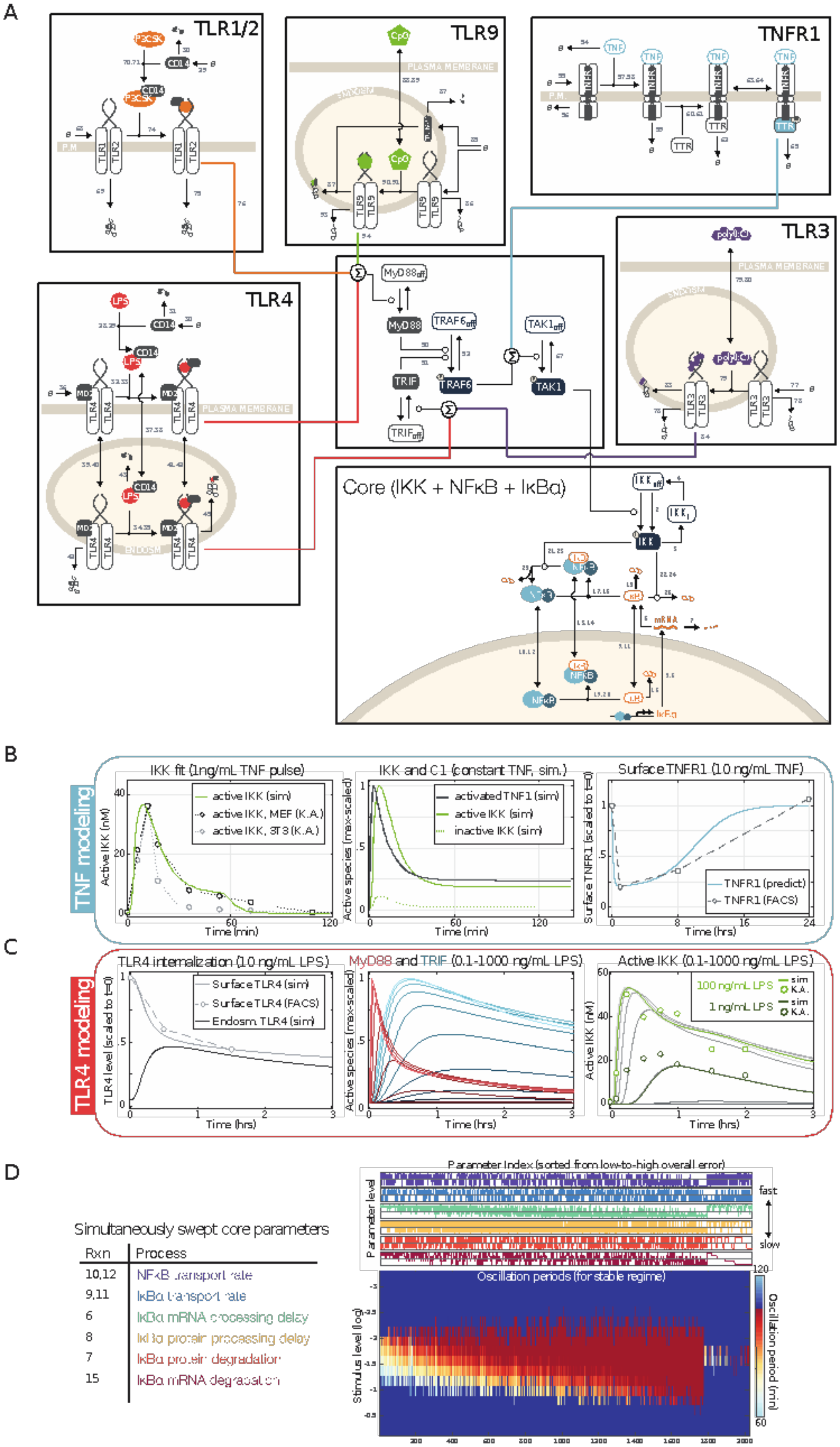
A
TLR1/2
TLR9
TNFR1
TLR3
TLR4
PLASMA MEMBRANE
ENDOSOME
Core (IKK + NFκB + IκBα)
B
TNF modeling
IKK fit (1ng/mL TNF pulse)
active IKK (sim)
active IKK, MEF (K.A.)
active IKK, 3T3 (K.A.)
Active IKK (nM)
Time (min)
IKK and C1 (constant TNF, sim.)
activated TNF1 (sim)
active IKK (sim)
inactive IKK (sim)
Active species (max-scaled)
Surface TNFR1 (10 ng/mL TNF)
TNFR1 (predict)
TNFR1 (FACS)
Surface TNFR1 (scaled to t=0)
Time (hrs)
C
TLR4 modeling
TLR4 internalization (10 ng/mL LPS)
Surface TLR4 (sim)
Surface TLR4 (FACS)
Endosm. TLR4 (sim)
TLR4 level (scaled to t=0)
MyD88 and TRIF (0.1-1000 ng/mL LPS)
Active IKK (0.1-1000 ng/mL LPS)
100 ng/mL LPS
1 ng/mL LPS
sim
K.A.
Active IKK (nM)
D
Simultaneously swept core parameters
Rxn | Process
10,12 | NFκB transport rate
9,11 | IκBα transport rate
6 | IκBα mRNA processing delay
8 | IκBα protein processing delay
7 | IκBα protein degradation
15 | IκBα mRNA degradation
Parameter Index (sorted from low-to-high overall error)
Parameter level
fast
slow
Oscillation periods (for stable regime)
Stimulus level (log)
Oscillation period (min)

Figure S6. Constructing and fitting a mathematical model of NFκB activation dynamics in response to multiple stimuli, Related to Figure 6.

(A) Reaction schema for the multi-stimulus model of NFκB activation. Each box represents a regulatory module, with receptor-associated modules connecting into common core modules. All reactions are shown with numbers representing kinetic rate constants identified in Table S7.

(B) TNFR-associated module: Left: IKK activation in response to a 45 min pulse of TNF activation were fit using a screen where repeated, random initialization was followed by optimization: the best fit model of 1024 trials is shown. Middle: dynamics of TNFR1 and IKK activation. The transience of IKK activation is likely to be driven by rapid receptor internalization, not IKK inactivation, as has been previously hypothesized. Right: predicted levels of TNFR1 internalization in response to 10 ng/mL TNF, and measured surface TNFR1 levels as measured by FACS.

(C) TLR4-associated module: Left: dynamics of TLR4 internalization. TLR4 is also rapidly internalized in response to binding of LPS but stays active in the early endosome. Middle: dynamics of MyD88 and TRIF activation in response to 0.1 (dark curves) to 1000 (bright curves) ng/mL LPS. Right: fitted levels of IKK activation (note that peak activation is only app. 25% greater than activity induced by TNF) in response to 0.1 (dark curves) to 1000 (bright curves) ng/mL LPS. Simulated and measured (by kinase assay) dynamics at 1 ng/mL and 100 ng/mL LPS are highlighted in green.

(D) Results of a simultaneous parameter sweep in the "core" NFκB model (IKK, NFκB, and IκBα). Swept parameters are indicated in table on left. 1728 out of 2000 parameter combinations showed activation in response to a range of IKK values. The full dose response was measured and ranked along the oscillatory characteristics to ensure that parameter sets are robust in this key characteristic.

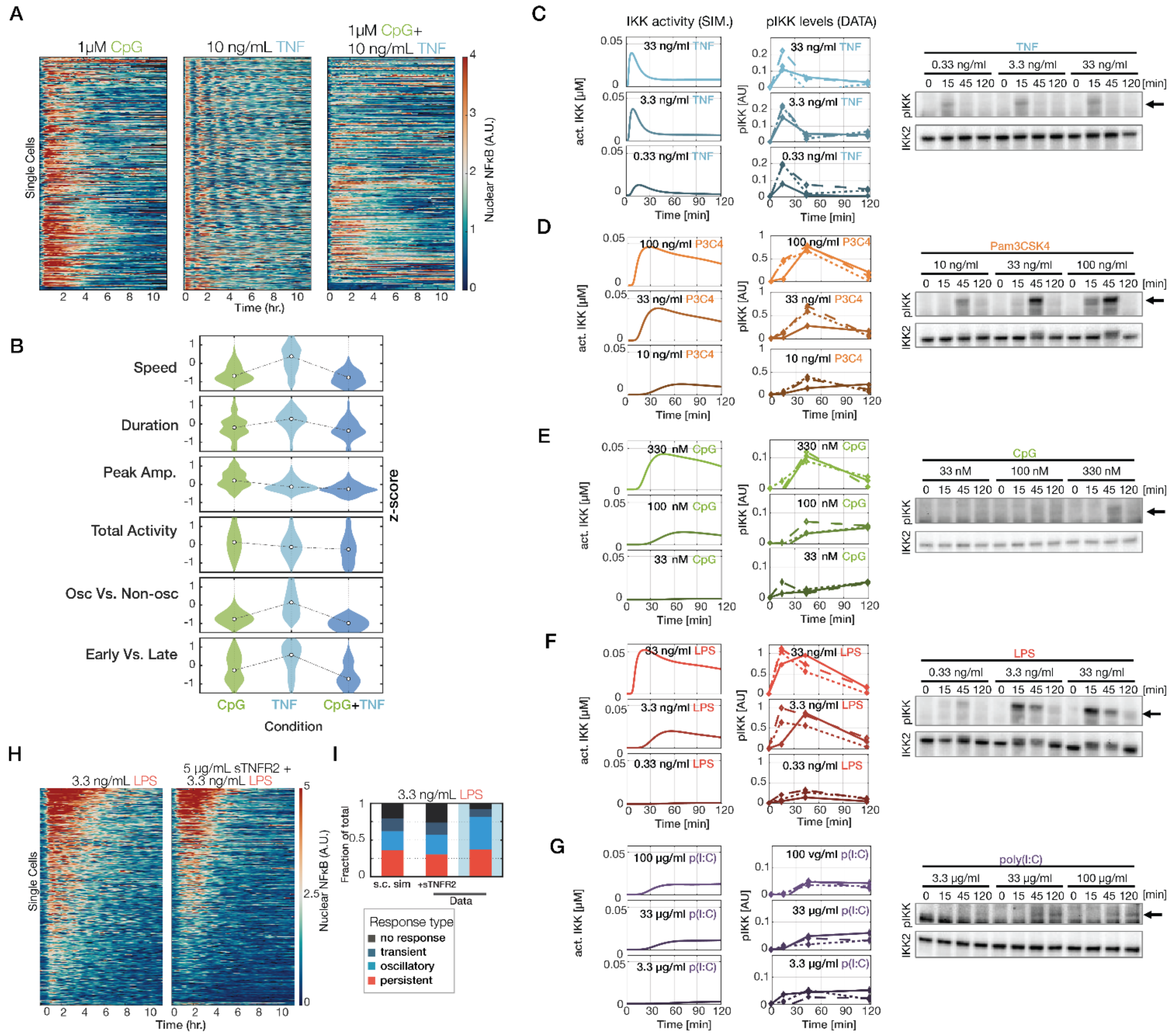

Figure S7. Distinguishing mixed ligands and IKK activation kinetics in primary macrophages, Related to Figure 7.

(A) Heatmaps of 1 µM CpG, 10 ng/mL TNF, and 1µM CpG + 10 ng/mL TNF stimulation.

(B) Deployment of signaling codons in mixed (CpG+TNF) stimulus compared to individual stimuli. Violin plots of the relative presence of indicated signaling codons (z-score) in the trajectories of individual cells.

(C-G) Levels of IKK activity in response to (C) TNF, (D) Pam3CSK4, (E) CpG, (F) LPS, and (G) poly(I:C) at the indicated doses. Left: Simulated IKK activity at 0 min to 120 min upon stimulation. Right: Western blots of phosphorylated IKK (pIKK) in lysates of BMDMs stimulated with indicated ligands, doses, and for indicated times. For quantification, pIKK band intensities were normalized to total IKK2 levels and scaled between baseline (set to 0) and a positive

control sample (set to 1; 33 ng/ml LPS, 45 min, from replicate 1). Quantifications from three replicates are shown (line styles indicate different replicates).

(H) Single-cell heatmaps of NFκB activation mVenus-RelA BMDMs in response to 3.3ng/ml LPS, with or without feedforward TNF signaling blocked using saturating amounts (5 µg/mL) of soluble TNFRII co-treatment.

(I) Proportions of NFκB dynamic subtypes (off, transient, oscillatory, or persistent) as quantified from the data in (H).
